# Hybrids of RNA viruses and viroid-like elements replicate in fungi

**DOI:** 10.1038/s41467-023-38301-2

**Published:** 2023-05-05

**Authors:** Marco Forgia, Beatriz Navarro, Stefania Daghino, Amelia Cervera, Andreas Gisel, Silvia Perotto, Dilzara N. Aghayeva, Mary F. Akinyuwa, Emanuela Gobbi, Ivan N. Zheludev, Robert C. Edgar, Rayan Chikhi, Massimo Turina, Artem Babaian, Francesco Di Serio, Marcos de la Peña

**Affiliations:** 1 Institute for Sustainable Plant Protection, National Research Council of Italy, Torino, Italy; 2 Institute for Sustainable Plant Protection, National Research Council of Italy, Bari, Italy; 3 Instituto de Biología Molecular y Celular de Plantas, Universidad Politécnica de Valencia-CSIC, Valencia, Spain; 4 Institute of Biomedical Technologies, National Research Council of Italy, Bari, Italy; 5 International Institute of Tropical Agriculture, Ibadan, Nigeria; 6 Department of Life Science and Systems Biology, University of Torino, Torino, Italy; 7 Institute of Botany, Ministry of Science and Education of the Republic of Azerbaijan, Baku, Azerbaijan; 8 Department of Agroforestry Ecosystems, Universidad Politécnica de Valencia, Valencia, Spain; 9 Department of Land, Environment Agriculture and Forestry, Università Degli Studi di Padova, Padova, Italy; 10 Department of Molecular and Translational Medicine, University of Brescia, Brescia, Italy; 11 Department of Biochemistry, Stanford University, Stanford, CA USA; 12 Independent Researcher, Corte Madera, CA USA; 13 G5 Sequence Bioinformatics, Department of Computational Biology, Institut Pasteur, Paris, France; 14 Institute for Sustainable Plant Protection, National Research Council of Italy, Brescia, Italy; 15 Department of Molecular Genetics, University of Toronto, Toronto, ON Canada; 16 Terrence Donnelly Centre for Cellular & Biomolecular Research, University of Toronto, Toronto, ON Canada; 17 Present Address: Department of Entomology and Plant Pathology, Auburn University, Auburn, AL USA

**Keywords:** Viral evolution, Molecular evolution, Fungi, Ribozymes

## Abstract

Earth’s life may have originated as self-replicating RNA, and it has been argued that RNA viruses and viroid-like elements are remnants of such pre-cellular RNA world. RNA viruses are defined by linear RNA genomes encoding an RNA-dependent RNA polymerase (RdRp), whereas viroid-like elements consist of small, single-stranded, circular RNA genomes that, in some cases, encode paired self-cleaving ribozymes. Here we show that the number of candidate viroid-like elements occurring in geographically and ecologically diverse niches is much higher than previously thought. We report that, amongst these circular genomes, fungal ambiviruses are viroid-like elements that undergo rolling circle replication and encode their own viral RdRp. Thus, ambiviruses are distinct infectious RNAs showing hybrid features of viroid-like RNAs and viruses. We also detected similar circular RNAs, containing active ribozymes and encoding RdRps, related to mitochondrial-like fungal viruses, highlighting fungi as an evolutionary hub for RNA viruses and viroid-like elements. Our findings point to a deep co-evolutionary history between RNA viruses and subviral elements and offer new perspectives in the origin and evolution of primordial infectious agents, and RNA life.

## Abstract (web summary)

RNA viruses are defined by linear RNA genomes encoding an RNA-dependent RNA polymerase, while viroid-like elements consist of small, single-stranded, circular RNA genomes that, in some cases, encode self-cleaving catalytic RNAs. Here, the authors identify over 20,000 candidate viroid-like elements, and show that infectious agents of fungi display hybrid features of viroid-like RNAs and RNA viruses.

## Introduction

Serving dual functions as encoding for genetic information, and as a biological catalyst, RNA has been proposed to predate DNA and protein at the origin of life in an “RNA World”^1–3^. Further, extant sub-viral and viral agents have been suggested to be “living fossils” that can help illuminate the molecular biology of Earth’s first life^4,5^.

Viruses having RNA genomes (realm *Riboviria*) are infectious agents defined by a linear RNA genome encoding one of their hallmark replication polymerases (replicases), either an RNA-dependent RNA polymerase (RdRp) for RNA viruses, or a Reverse Transcriptase (RT) for retroviruses. These replicases, and thus *Riboviria*, are monophyletic in nature as based on alignment of the conserved palm domain of RdRp and RT^6^.

**Fig. 1:**
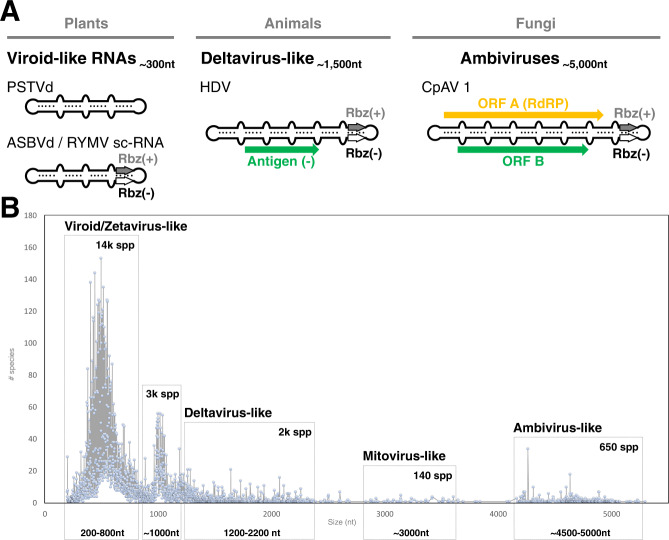
Subviral and ambivirus circRNA genome organizations and size distribution of the circRNA genomes with two ambisense ribozymes. **A** Genome organization of representative sub-viral and viral agents with genomes of circRNA: non-coding viroids and viroid-like satellite RNAs from plants; antigen protein-coding deltavirus-like from animals (*Ribozyviria*) and RdRp-coding ambiviruses from fungi. **B** Distribution graph of the 20,364 detected species-like operational taxonomic units (sOTU) at 90% nucleotide sequence identity based on their nucleotide length. Approximated number of sOTU are indicated within each of the 5 major groups: small or viroid/Zetavirus-like^14^ (200–800 nt, 14,526 species), medium (800–1200 nt, 3233 species), medium-large or deltavirus-like (1200–2200 nt, 1951 species), large or Mitovirus-like (around 3000 nt, 147 species) and very large or Ambivirus-like (around 5000 nt, 653 species).

Viroids and viroid-like entities (such as the human satellite virus, hepatitis Delta virus), are infectious sub-viral RNA agents of plants and animals (Fig. 1A). These sub-viral agents are typified by small (~200–1800 nt), single-stranded, circular RNA (circRNA) genomes which form extensive secondary structures, typically rod or branched-rod folding. Some of them encode hallmark paired self-cleaving catalytic RNAs or ribozymes, one in each strand polarity. These autocatalytic ribozymes are structural RNA elements that, in the absence of proteins, carry out site-specific phosphodiester scission of the oligomeric RNAs generated during the replication of the circRNA genome through a rolling-circle mechanism^4,5^. So far, the ribozymes reported in infectious circRNAs are hammerhead (HHRz)^7^, hairpin (HPRz)^8^ and hepatitis Delta (DVRz) ribozymes^9,10^. In contrast to RNA viruses, infectious circRNAs replicate by RNA polymerases encoded in *trans* by the host (viroids and hepatitis Delta viruses) or helper viruses (satellite circRNAs). Additionally, while viroids are non-coding RNAs^4^, deltavirus-like RNAs code for the conserved Delta Antigen proteins and have been assigned the realm *Ribozyviria*^11^. Overall, the simple genomes and small ribozymes of infectious circRNAs are why these elements are regarded as molecular fossils of the prebiotic world^4^.

Ambiviruses are a recently characterized and widespread group of fungal single-stranded RNA infectious agents with a bicistronic, ambisense genome of ~5 kb encoding for two conserved open reading frames (ORFs) A and B, one in each polarity strand (Fig. 1A). ORF-A has a remote similarity to viral RdRps leading to these agents being reported as RNA viruses. However, northern blotting, RT-PCR, and de novo transcript assembly indicated contiguity between 3’ and 5’ terminal ends, inconsistent with the assumed typical linear RNA virus genome^12,13^.

Here we report that the number of potential new viroid-like circRNAs occurring in geographically and ecologically diverse niches is much higher than previously thought. Among them, we chose to investigate the most fascinating elements, the ambiviruses. We show that they have circRNA genomes and encode paired self-cleaving ribozymes active in vivo, enabling a rolling circle mechanism of replication, the typical features of viroid-like RNAs reported so far. However, we further show that ORF-A has the typical features of a functional RdRp, like that of a classical RNA virus. Thus, ambiviruses appear to have likely arisen from the genetic recombination of a viroid-like genomic backbone with the hallmark RNA virus gene RdRp, and as such are an intermediate class of infectious RNAs. This finding bridges the RNA virospheres of viruses and viroids, posing deep questions regarding their origins and (co-)evolution.

## Results

### Ultra-high-throughput screen to identify novel subviral agents

To screen for potential new ribozyme-bearing subviral agents, we adapted the *Serratus* ultra-high-throughput computing architecture previously used for RNA virus discovery^14^ to search for ribozymes using INFERNAL across 198,194 raw metagenome/metatranscriptomes freely-available in the Sequence Read Archive^15,16^. Combining the top 5000 ribozyme-hit libraries with our previous RNA virus assemblage and the Transcriptome Shotgun Assembly (TSA), we created the “RNA Deep Virome Assemblage” (RDVA) of 58,557 libraries. From the 12.5 billion assembled contigs, 34 million contained 5’–3’ k-mer overlaps consistent with a circular molecule. Filtering the potential circular contigs further for those encoding both a plus-sense ribozyme and a negative-sense (paired-ribozymes), resulted in a discovery set of 32,393 ribozyme-bearing potentially circular elements, which clustered into 20,364 units at 90% nucleotide sequence identity. Based on their sizes, the vast majority of the clusters group into distinct populations of either small- (70% are 200–800 nt long) or medium-size genomes (25% are 800–2 kb) (Fig. 1B and Supplementary Fig. 1), which show features reported for viroid- and deltavirus-like genomes, respectively: circularity, predicted stable secondary structures (either rod-like or branched), two ambisense ribozymes and diverse protein-encoding capabilities^4^. As recently described^14,17^, most of these RNAs occur in environmental metatranscriptomics in which host inference is difficult. However, our analysis of runs from a collection of isolates of *Rhizoctonia solani* and *Tulasnella* spp. revealed 7 examples of viroid- and deltavirus-like genomes, which were confirmed to exist solely as RNA species and, most likely, are novel infectious subviral agents (Supplementary Fig. 2). Interestingly, a significant fraction of all the detected circular genomes (~4%) however, shows much larger sizes (~3–5 kb) out of the typical length of subviral agents of circRNA.

### Ambiviruses have circRNA genomes with ribozymes

Analysis of large ribozyme-bearing circRNAs (~5 kb) unveiled sequence similarity with diverse ambivirus genomes, suggesting that their unusual features derived from 5’ RACE analyses could be due to a circular genome (Supplementary Note 1 and Supplementary Fig. 3). Moreover, we analyzed published ambivirus genomes for sequence homology to structured RNA covariance models with INFERNAL^18^. This uncovered head-to-tail oriented, ambisense HHRz or HPRz motifs in most of the GenBank sequences, reminiscent of subviral circRNAs (Supplementary Table 1).

**Fig. 2:**
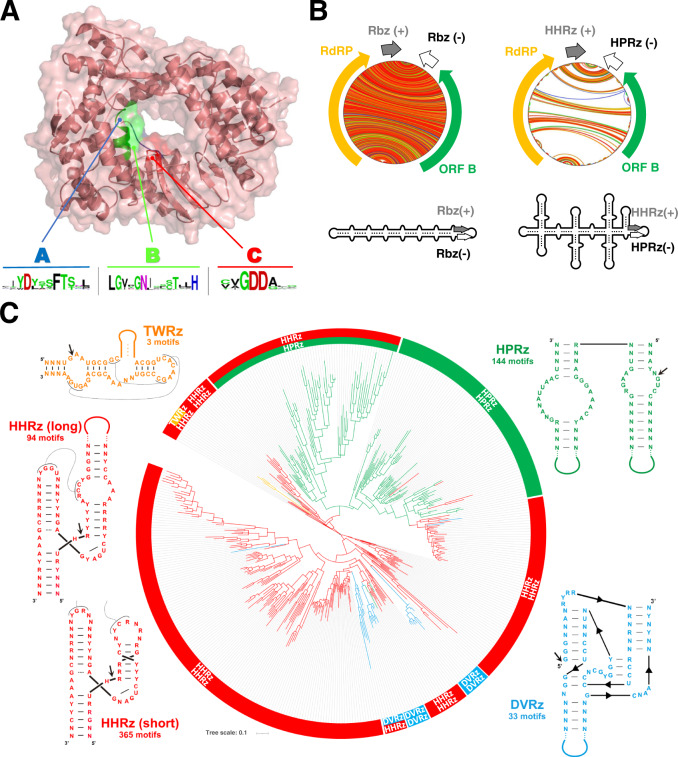
Ambivirus RNA-dependent RNA polymerase phylogeny. **A** ColabFold-AlphaFold2 predicted structure of the representative Armillaria borealis ambivirus 2 (Accession MW423810.1) ORF-A shows a classic polymerase palm fold structure. Conservation of catalytic residues in motif A, B, and C is seen across all ambiviruses identified in this study and present in databases. **B** Circular plots of the predicted RNA secondary structures of two representative ambiviruses. On the left, an example of the characteristic rod-like structure predicted for most ambiviruses. On the right, an example of a highly branched architecture predicted for some ambivirus RNA genomes, usually carrying a mixed pair of HHRz/HPRz motifs. **C** Maximum-likelihood phylogenetic tree of the RNA-dependent RNA polymerase palmprints from the 439 distinct species-like operational taxonomic units (sOTUs). A more detailed view of the tree can be found at the Supplementary Fig. 7. There are up to six major clades of ambiviruses showing diverse ribozyme usage. Up to 60% sOTUs use paired HHRz very similar to the motifs from Epsilonviruses^14,41^ or plant viroids^4^, whereas ~30% carry either two HPRz or a HHRz/HPRz mix, 5% carry the DVRz (either paired or a mix with HHRz) characteristic of animal deltaviruses, and three sOTUs contain a mix of the TWRz (P1 architecture)^42^ and the HHRz motifs. Consensus ribozyme structures (weighted nucleotide conservation threshold of 70%) of twister (TWRz, in orange), the hammerhead (HHRz, in red, two length variants), hairpin (HPRz, in green) and Delta virus (DVRz, in blue) ribozymes present in ambiviruses are shown. Site of self-cleavage is indicated with an arrow.

The initially reported similarity between ambivirus ORF-A and RdRp was not statistically significant owing to their deep divergence^12,13^. However, searches for ambiviral RdRps in the RDVA and other public databases (see Methods) revealed up to 974 unique RdRp-encoding ambiviruses, which clustered into 439 sOTUs at 90% palmprint amino acid identity^19^. Again, these genomes were found widespread in diverse environmental datasets (Supplementary Fig. 4). This high number of ambivirus RdRp sequences provided us the opportunity of further exploiting the in silico structural prediction that have extended the capacity to assess for deep protein homology from sequences alone^20^. ColabFold-AlphaFold2 prediction of ORF-A supports (pLDDT confidence up to 93.5 for several models) that these proteins take on the classic right-handed palm domain architecture, with highest structural similarity to negarnavirus RdRps (Influenza A, *Z*-score >19, RDSM 3.8 A) (Supplementary Fig. 5). Critically, the expanded multiple sequence alignment shows sequence conservation across all the essential polymerase catalytic motifs A, B, C, D, E and F^19^ (Fig. 2A and Supplementary Fig. 6). In addition, prediction of the secondary structures of minimal free energy for all the ambiviral potential circular genomes revealed that the majority (90%) of these RNAs adopt a rod or quasi-rod like structure of very high stability, akin to most plant viroids and Delta-like viruses. However, about 10% of the ambivirus genomes adopt a highly branched RNA conformation (Fig. 2B), suggesting that extensive genomic base-pairing structural constraints are preserved across ambiviruses.

Reconstruction of phylogeny shows a complex natural history of the ambivirus RdRp and their ribozymes. Four out of the nine known self-cleaving ribozymes^21^ were found in ambivirus genomes, thereby suggesting that multiple recombination and/or horizontal transfer events of ribozymes have likely occurred (Fig. 2C and Supplementary Fig. 7). Intriguingly, there is also extensive mixture of two different ribozymes on a single genome. Similar ribozyme mixtures are observed in the 5% of our discovery set of small viroid-like genomes, supporting that different ribozyme classes may coexist within a genome.

### Molecular validation of circularity and autocatalysis of the ambivirus genomes

**Fig. 3:**
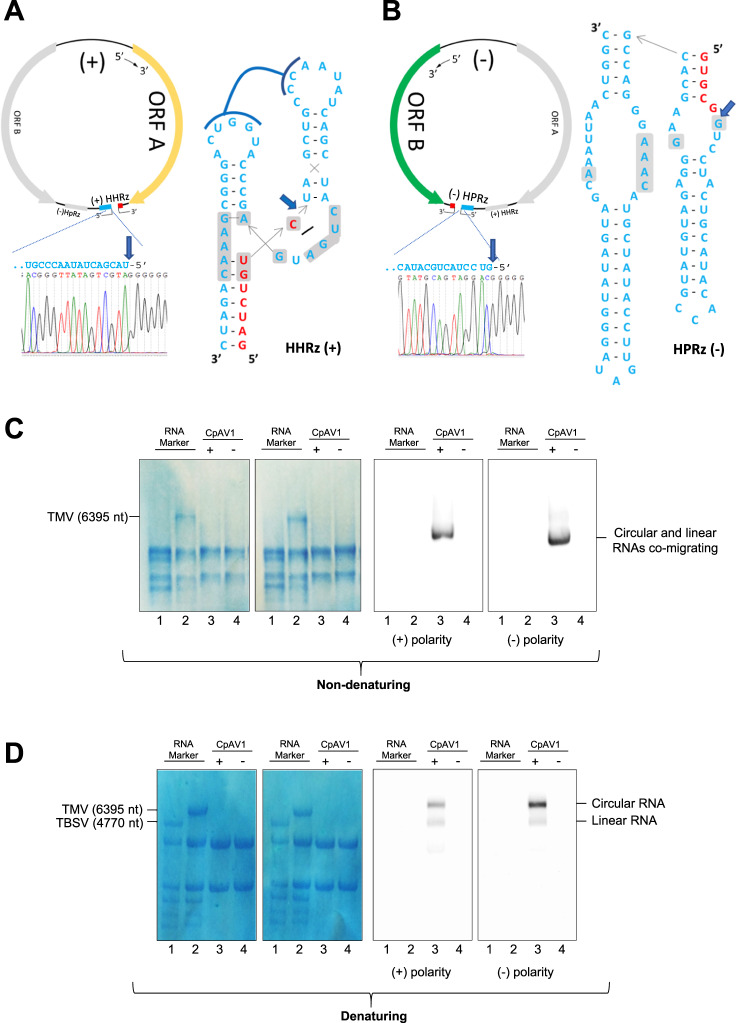
Ambiviruses encode functional +/− strand ribozymes and have circRNA genomes. 5’ RACE and Sanger sequencing of the (**A**) positive and (**B**) negative strand of TuAmV4 genomic RNAs show in vivo self-cleavage (blue arrow) at the predicted cut-sites for its HHRz and HPRz motifs, respectively. Ribozyme secondary structures drawn as inlay. **C**, **D** Northern blot assays of RNA preparations separated by electrophoresis under non denaturing (**C**) and denaturing (**D**) conditions and hybridized with probes specific for the (+) and (−) CpAV1 RNA; Lanes 3 and 4 are RNAs from CpAV1-infected and non-infected *Cryphonectria parasitica*, respectively; RNA markers are RNAs from plants, healthy or TBSV-infected (line 1 in **C** and **D**, respectively) and TMV-infected (line 2 in **C** and **D**). The single signal detected in the infected isolate with the either probe under non-denaturing conditions corresponds to both circular and linear CpAV1 genomic RNAs (4663 nt), which are expected to co-migrate (**C**). In denaturing conditions (**D**), the ambivirus circRNAs are delayed with respect to the respective linear forms (lower band). In **C**, **D**, the two leftmost panels are total RNA stained with methylene blue. The same results were obtained from three independent experiments.

To validate this in silico expansion of ambiviruses, we molecularly evaluated key properties from the predicted sequences in selected fungal isolates. Self-cleavage of ambivirus ribozymes was confirmed by in vitro transcription of their cDNAs from Cryphonectria parasitica ambivirus 1 (CpAV1) (two HHRzs), Tulasnella ambivirus 4 (TuAmV4) (one HHRz and one HPRz) and TuAmV1 (two HHRzs). For all tested sequences, transcripts showed self-cleavage, and termini of resultant fragments are consistent with the predicted ribozyme cleavage sites (Supplementary Fig. 8). To determine if cleavage occurred also in vivo, 5’ RACE was performed from infected *Tulasnella* spp. and *Cryphonectria parasitica* extracts, and again showed perfect agreement with in silico prediction and in vitro results (Fig. 3A, B and Supplementary Fig. 9).

Such functional self-cleaving ribozymes on both polarities are only known to occur in diverse viroid- and Deltavirus-like agents, all of which possess circRNA genomes which replicate, in the absence of any DNA counterpart, through a symmetric rolling circle mechanism. Another hallmark of symmetric rolling circle replication is the in vivo accumulation of circRNAs of both polarities^22^. To evaluate if ambiviruses have circRNA genomes, northern blotting against CpAV1 was done under native and denaturing conditions (Fig. 3C, D). Under native conditions, the CpAV1 genomic RNA migrated as a single band corresponding to the monomeric RNA (around the expected 4623 nt), while under denaturing conditions, two bands were resolved. The retarded band would correspond to a circular molecule, and indeed the upper band shows preferential resistance to RNase R exonuclease treatment (Supplementary Fig. 10). Together, this demonstrates that in vivo CpAV1, and by extension ambiviruses, replicate through a symmetric rolling circle mechanism.

Finally, to associate a phenotype to ambivirus infection, we obtained ambivirus-infected and ambivirus-free isogenic conidial isolate and show that CpAV1 causes hypovirulence in its fungal host (Supplementary Fig. 11), a feature that is useful for biocontrol of this important chestnut tree disease; this is the first example of a biotechnologically exploitable property linked to this new group of infectious agents.

### Mitochondrial viruses with self-cleaving genomes of circRNA

**Fig. 4:**
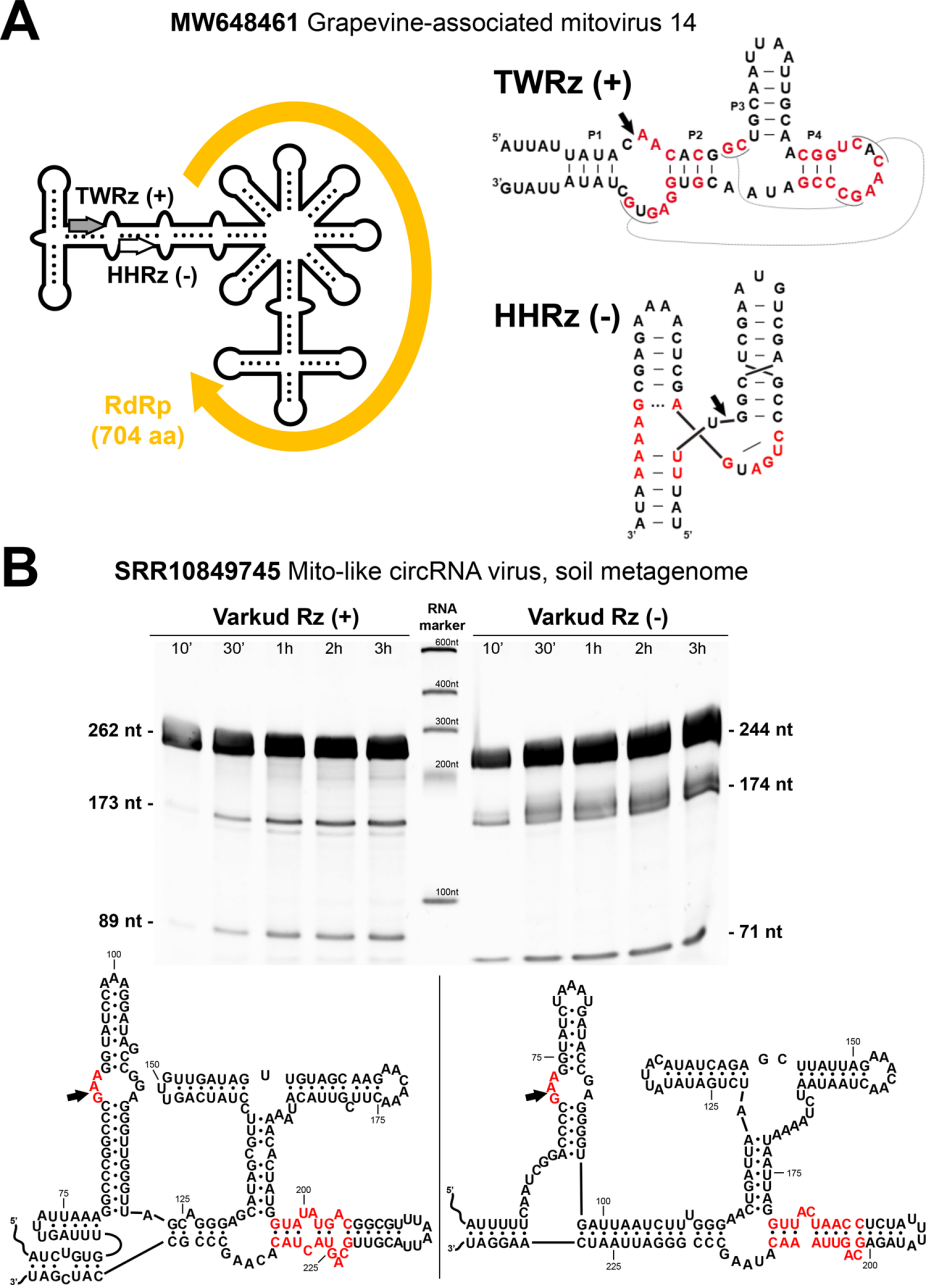
A further group of RdRp/ribozyme hybrid molecules. **A** Schematic genome organization of the Grapevine-associated mitovirus 14 (Accession MW648461.1). The deposited sequence contains a 203 nt repeat at both ends of the genome, indicative of a circRNA. The predicted ORF corresponding to the RdRp is flanked by a twister (TWRz) and a hammerhead (HHRz) ribozyme in the plus and minus polarities, respectively. **B** A mitovirus with a putative circular genome detected in the library SRR10849745 (soil metagenome) shows the presence of a Varkud Satellite ribozyme in each polarity. Denaturing PAGE of the RNA products from run-off transcriptions of both ribozyme constructs obtained at different incubation times shows the expected sizes of the primary and cleavage RNA products. Site of self-cleavage is indicated with an arrow, and conserved nucleotides for each ribozyme are shown in red.

To see if other “RNA viruses” share a viroid-like genomic backbone, we searched the RDVA set for contigs with dual polarity paired-ribozymes and identified up to 16 circular contigs of approximately 3 kb encoding RdRp-like ORFs (Supplementary Table 2 and Fig. 4) sharing sequence similarity to diverse mitovirus RdRps. Among them, we detected 7 novel circular genomes similar to *Fusarium asiaticum* mitoviruses, which all carry dual-polar variants of a rare and complex self-cleaving motif, the Varkud Satellite ribozyme (VSRz), so far only described in the mitochondrial VS plasmid of some *Neurospora* isolates^23^. The self-cleaving activity of mitovirus VSRzs was experimentally confirmed, whereas the predicted secondary structure of these agents was found to be highly branched (Fig. 4). These findings extend the presence of circular genomes with paired ribozymes to a different group of RNA mycoviruses and suggest that the emergence of agents showing hybrid features of viroid-like RNAs and viruses may have occurred multiple times in evolutionary history.

## Discussion

Infectious circRNAs have been regarded as biological oddities, with less than 50 accepted viroid-like species such as plant viroids and the human Deltavirus^24^. In this work we report more than 20 thousand potential viroid-like element species, advancing that they are more widespread than previously appreciated, and warrant further molecular characterization. These viroid-like elements can range from small viroid-like, medium sized delta-like, to large virus-like elements, which are hybrid infectious RNAs with circular genomes encoding autocatalytic ribozymes and a viral RdRp. Our findings offer a new viewpoint to the traditional distinction between viruses and viroid-like RNAs based on the respective linear and circular genomes, encoding or not their own replicase. Ambiviruses and mitoviruses are two examples of such hybrid infectious agents that, besides expanding the host range of ribozyme-encoding circRNAs to fungi, extend the genome size of infectious ribozyme-encoding circRNAs up to 5 and 3 kb, respectively. Genome length expansions were favored by the incorporation of new genetic information into a small viroid-like RNA backbone which must overcome the added energetic cost of the resulting genome.

The identification of TWRzs and VSRzs in ambiviruses and mitoviruses, respectively, shows that the type of ribozymes involved in the replication of infectious circRNAs is not limited to HHRz, HPRz, and DVRz, as previously thought and suggests the possible involvement of other known or yet unknown types of ribozymes in the replication of some of the thousands new potential viroid-like circRNAs reported. The unexpected combination of different ribozymes in the same circular genome shows a frequent coexistence of different types of ribozymes, and suggests that horizontal transfer of genetic information may occur amongst viroid-like elements. Altogether these findings pose intriguing questions on the origin of these hybrid infectious agents, supporting a major role of recombination events between RNA modules providing self-cleaving activity in each polarity strand with genes encoding the needed replicase activity.

While computational advances will drive the exponential discovery of these elements (similar to RNA viruses)^14,25^, emphasis should be placed on collaborative molecular characterization of this new universe of infectious agents. While commonly we associate these entities as infectious agents of mitochondria (some mitoviruses), fungi (ambiviruses), plants (viroids), or animals (delta-like viruses), these still limited host-distributions likely reflect our ignorance of the full diversity of infectious circRNAs across all kingdoms.

Our in vivo demonstration that fungi harbor infectious circRNAs expands the experimentally validated hosts for viroid-like molecules beyond plants and animals to also include the kingdom fungi. With their multiple symbiotic relationship with plant and animal hosts, fungi are indeed a hub for generating trans-kingdom horizontal transfer of infectious elements such as those described here^26^. The phenotype associated to CpVA1 infection also hints at the possibility that these viroid-like infectious agents are new components of the holobiont and contribute to intraspecific phenotypic variability. Furthermore, these agents (ambivirus in specific) could develop into circRNA expression vectors, a further step into protein expression stabilization from RNA templates^27^.

Given the rapid mutation rate of viroids^28^, the disposition of viroid-like elements to swap ribozymes (as shown here), and the limitations of phylogenetic analysis, the deep evolutionary relationships between infectious circRNAs (such as the genomic origin of ambiviruses) is likely unknowable. Further, structurally simple ribozymes, such as HHRz can arise spontaneously in evolution^29^, as is the case in relatively simple head-to-head ligated RNAs, supporting the notion that the simplest classes of infectious circRNAs may be evolving de novo continuously. As viroid-like circRNAs can acquire genes, such as a delta-antigen or viral RdRp, and give rise to more complex entities, it follows that host RNA polymerases may have been exapted to give rise to Earth’s primordial “viruses”. Perhaps most tantalizingly, the acquisition of an early RNA replicase ribozyme would serve as a blueprint for the simplest replicator of the RNA world, and thus a major step in determining the origins of life^4^.

## Methods

### Ribozyme search of the Sequence Read Archive

Observing that ribozymes are sufficiently short to be captured on a short sequence read (less than 100 nt), we reasoned it will be possible to screen large volumes of sequencing data to identify libraries potentially containing ribozyme agents. To this end we adapted the ultra-high throughput Serratus cloud computing platform^14^, with the INFERNAL^18^, an algorithm for identifying RNA elements using secondary structure and nucleotide covariation modelling. Using Serratus-INFERNAL (https://github.com/ababaian/serratus/tree/infernal-dev), we performed a screen of 198,194 publicly available metagenomic/metatranscriptomic sequencing libraries in the Sequence Read Archive (SRA) for the delta, hairpin, hammerhead, twister, and Varkud satellite ribozymes (hatchet, pistol, twister sister and glmS ribozymes were discarded due to the absence of hits in previous analyses). The SRA search query was randomly sub-sampled from the set of “METAGENOME” OR “METATRANSCRIPTOME” OR “metatranscriptomic”[Filter] OR “metagenomic”[Filter] NOT amplicon [All Fields] AND “platform illumina”[Properties] AND cluster_public[prop]” on 2022-01-13. We then heuristically rank ordered the libraries based on the quality and number of distinct ribozyme hits in a library as well as sequencing depth for focused sequence assembly of the SRA. Candidate viroid-like and ribozyviria sequences were identified by the following criteria, all of which must be satisfied: (1) the presence of 5’–3’ end k-mer overlaps suggestive of a circular molecule, (2) a pair of complementary hits to one ribozyme model with *E*-value <1e−3, and (3) <25% repetitive sequence according to the union of hits reported by dust^30^, Tandem Repeat Finder^31^ and self-hits according to ublast^32^. Clustering into 90% identity species-like Operational Taxonomic Units was performed using circuclust (https://github.com/rcedgar/circuclust).

### Bioinformatic detection and phylogeny of ambiviral RdRps

RdRps (ORF-A) from either the 19 deposited ambivirus genomes at the GenBank (09-2021), or the 169 ambiviral-like sequences (either putative full circles or genomic fragments) detected though INFERNAL in public metatranscriptomic datasets (“metatranscriptome” OR “metatranscriptomic”[Filter]), were used to build a consensus sequence for the ambivirus motifs A, B and C. Based on the obtained consensus sequence for the ambiviral palm domain, we built a new version of the palmscan software (https://github.com/rcedgar/palmscan) capable of ambivirus RdRp detection. Palmscan searches were performed in the circular contigs from the RDVA, which together with the ones obtained from GenBank resulted in 439 ambiviral RdRps at 90% sequence identity (https://github.com/ababaian/serratus/wiki/ambivirus_extended_data/). We performed multiple sequence alignment on the clustered RdRp palmprint sequences using MUSCLE^33^ and maximum-likelihood tree is inferred from the alignment using MEGA11^34^. Secondary RNA structures of minimum free energy of the ambivirus genomes were calculated with the RNAfold program from the ViennaRNA Package^35^. The obtained structures were visualized by circular plots obtained with the jupiter software (https://github.com/rcedgar/jupiter).

### Fungal growth in liquid media

To obtain fungal mycelia for molecular analysis, three CpAV1-free and three CpAV1-infected isolates were inoculated in liquid media in biological triplicate (18 samples). The media contained a mix of 24 g/L potato dextrose broth (PDB) (SIGMA) with two European chestnut (*Castanea sativa*) sticks of 8 cm length (cut longitudinally) autoclaved at 121 °C for 1 h. 50 mL of the mix was transferred into 100 mL conical flasks for all the samples. Then, 12.5 mL of the liquid media was collected in 50 mL conical Falcon^TM^ tubes (Corning^TM^ Fisher Scientific) where 30 mycelia plugs were transferred for each of the replicates. The tissues were ruptured with Omni Tissue Homogenizer (Qiagen) for 1 min and 3.5 mL of the homogenized paste was inoculated in each of the conical flasks containing the liquid media. The cultures were kept constantly shaking at 120 rpm at room temperature.

At three days post-inoculation, the fungal cultures were harvested by filtering with a Buchner funnel covered with one layer of Miracloth (Calbiochem) connected to a vacuum pump to separate the mycelia from the liquid media; the harvested mycelia were frozen at −80 °C and lyophilized for 45 h (EDWARDS MODULYO freeze dryer). Mycelial dry weight for each replicate was determined with a top pan balance.

*Tulasnella* spp. isolates were grown in liquid cultures and harvested and lyophilized as above.

### RNA extraction

RNA extraction was done with Spectrum Plant Total RNA kit (SIGMA-Aldrich) following the manufacturer’s protocol with minor modifications: briefly, lyophilized mycelia (100 mg) was broken in 1.5 mL conical microcentrifuge tubes with O-rings (Biosigma) with 10, 4.5 mm diameter, ceramic beads in a FastPrep-24^TM^ homogenizer from MP Biomedicals^TM^ for 30 seconds at setting 6. A 1 mL mixture of lysis buffer and 2-mercaptoethanol (Sigma-Aldrich) at 1:100 volume dilution was added to the samples and mycelia was extracted by another round of bead beater, as above; the sample was then centrifuged at 10,000 × *g* for 4 min. 700 μL of the supernatant was then transferred to filter column tubes provided in the kit and centrifuged for 30 s at the same speed. The columns were discarded and 700 μL of the binding solution was added to the flow-through. This mixture was then passed through the binding column by a further centrifugation as above. The column was collected, transferred to a fresh collection tube and washed twice with wash buffer I and II (600 and 700 μL, respectively). RNA was then eluted in a 40 μL elution buffer. RNA quantification was done with Nanodrop LITE Spectrophotometer (Thermo Fisher Scientific).

### Characterization of terminal sequences of ambivirus genomes in vivo

Rapid Amplification of cDNA Ends (RACE) analysis was performed on CpAV1 from *C. parasitica* strain ACP34^13^ and TuAmV1 and TuAmV4 from Tulasnella spp. (respectively from strains MUT4048 and MUT4047)^12^ using the Hirzmann method^36^. Briefly, cDNA of the 5’ end of the positive and negative sense of the ambivirus genome was synthesized with the First Strand cDNA synthesis Kit #K1612 from Thermo Scientific with a specific primer (Supplementary Data 1) following the manufacturer’s protocol, and cleaned with Zymo clean and concentrator 5 kit (Zymo research, Irvine, CA, USA) and treated with Terminal Transferase (NEB, Ipswich, MA, USA) to add a poly-A or poly-G tail. After terminal transferase reaction, the cDNAs were cleaned using DNA Clean & Concentrator^TM^ 25 (Zymo research, Irvine, CA, USA) with a final elution volume of 10 µL and 1 µL of the obtained cDNAs was used as template to perform a PCR reaction with Taq polymerase (OneTaq kit from Biorad) following manufacturer’s protocol using a specific primer and anchored poly-T or poly-C primer complementary to the added tail at 51 °C in the annealing step for 30 s, 15 s at 68 °C for extension step and 98 °C for the denaturation step for 15 s. The resulting amplicons were fractionated by gel electrophoresis in 1% Agarose 1× TAE buffer and purified using a Zymo gel DNA recovery kit (Zymo research, Irvine, CA, USA) and cloned in pGEM-T easy vector system (Promega, Madison, WI, United States) following manufacturer’s instructions. Ligation products were transformed in DH5 alpha *E. coli* cells prepared and transformed following exactly the protocols and materials of the Mix&Go! *E. coli* Transformation Kit (Zymo Research). Sanger sequencing of the obtained clones (at least 3 clones containing the terminal region, for each construct) was performed by Biofab research (Rome, Italy) using the specific primers detailed in Supplementary Data 1.

Attempts were performed to directly sequence the 3’ end of both the positive and negative sense of the CpAV1 and TuAmV1 ambivirus genome by a method modified from Lambden et al.^37^ which is optimized for the sequencing of double-stranded RNA, but all failed. Briefly, a 5’-phosphorylated, 3’-amino-linked oligodeoxy-nucleotide primer was ligated to the 3’ termini of the total extracted RNA (1 µg total RNA) with T4 RNA ligase at 4 °C overnight. The ligated RNA was cleaned in column (binding column of the Spectrum Plant Total RNA kit, SIGMA), the cDNA was synthetized (Superscript IV, Thermo-Fisher) with a primer complementary to the ligated adaptor, cleaned using Zymo clean and concentration 25 kit (Zymo research, Irvine, CA, USA) with a final elution volume of 25 µL; 1 µL of the obtained cDNAs was used to perform a PCR reaction using a specific primer and the primer used for the cDNA synthesis with the same conditions described above, except the annealing temperature at 55 °C.

### Analyses of RNA self-cleavage in vitro

Self-cleavage activity of HHRz and HPRZ of representative ambiviruses (CpAV1, TuAmV1 and TuAmV4) and VSRz of the mito-like virus detected in the SRR10849745 library was tested in vitro by transcription of recombinant plasmids containing the cDNA of the respective ribozymes. The cDNAs of viral RNA fragments containing the ribozymes were either amplified using the appropriate primers listed in Supplementary Data 1 or purchased as gBlock Gene Fragments (Integrated DNA Technologies), subcloned in either pBlueScript KS or pGEM-T easy Promega, (Madison, WI, United States) and sequenced to confirm the orientation and absence of unwanted mutations. The recombinant plasmids, linearized with the appropriate restriction enzyme, were transcribed in vitro with T7 RNA polymerase (Thermo-Fisher Scientific) at 37 °C for 2 h using buffer provided by the manufacturer and the reaction products separated by denaturing 5% PAGE 8 M urea and 1× TBE (89 mM Tris, 89 mM boric acid, 2.5 mM EDTA, pH 8.3), stained with ethidium bromide and UV visualized. The 5’ terminal nucleotide of the 3’ self-cleavage product of HHRz and HPRz motifs was determined by 5’ RACE experiments. Briefly, the 3’ RNA fragment generated by the self-cleavage of each tested ribozyme during the in vitro transcription was eluted from the gel by grinding with a mixture (1:1) of water-saturated phenol and buffer (100 mM Tris-HCl pH 8.9, 1 mM EDTA, 0.5% SDS), and recovering the nucleic acid from the aqueous phase by ethanol precipitation^38^ then their 5’ terminal sequence was determined by 5’RACE as described above using the primers indicated in Supplementary Data 1.

### Northern blot hybridization assays

Agarose (1%) gel electrophoresis under denaturing conditions of total RNAs was performed using 6 M glyoxal and 50% v/v DMSO in HEPES EDTA buffer, keeping the RNA samples at 55 °C for 20 min prior to loading, following the protocol described previously^12^. Agarose gel electrophoresis of RNA preparations under non-denaturing conditions was carried out in 1× TAE without denaturation prior to loading.

After electrophoresis, RNA was blotted on Immobilon-Ny+ membrane (Merck, Darmstadt, Germany) and crosslink was performed in a Biorad gs gene linker UV chamber (Bio-Rad, Hercules, CA, USA). The membranes were hybridized with digoxigenin (DIG)-labeled riboprobes specific for (+) or (−) polarity strands of CpAV1 that were synthesized by in vitro transcription using the Dig-RNA labeling mix (Roche Diagnostics GmbH, Germany) and linearized plasmids containing CpAV1 cDNA inserts from positions 2798 to 3088 (located in ORFA) and from positions 1628 to 1998 (located in ORF B), respectively. Pre-hybridization and hybridization were performed in DIG easy Hyb (Roche Applied Science, Germany) buffer at 65 °C. After hybridization, the membranes were washed twice in 2× SSC (0.3 M NaCl, 0.03 M sodium citrate, pH 7), 0.1% SDS at room temperature for 10 min, and twice in 0.1× SSC, 0.1% SDS at 65 °C for 15 min. The hybridization signals were revealed with the anti-DIG alkaline phosphatase conjugate AB fragments and the chemiluminescence substrate CDP-star (Roche Applied Science, Germany) following the manufacturer’s instructions and visualized with a Chemidoc Touch Imaging system (Bio-Rad, Hercules, CA, USA).

### qPCR on the terminal sequences of the cleaved genome

In order to investigate whether the terminal fragments of the genomic and antigenomic strands of the ambivirus RNAs were fully complementary, we performed strand-specific qPCR analysis on TuAmV1 RNAs. cDNAs were synthesized from the same amount of RNA with primers specifically annealing either on the genomic or on the antigenomic strand (Supplementary Data 1) and designed either on the terminal putative sequence found by RACE, or on a region up/downstream. A pair of primers was designed on the ORF A and the qPCR product obtained was used as internal reference for the quantification of the terminal sequences. The cDNAs were column-purified (DNA Clean & Concentrator^TM^ 25 Zymo Research) and a standard qPCR was performed using the same primers: briefly, qPCR reaction was performed in 10 μL final volume using the SYBR® Green Master Mix (Biorad). Each 10 μL reaction mix contained 5 μL mix, 4 μL of sterile water and 0.2 μL of forward and reverse primers (Supplementary Data 1), and 1 μL of cDNA. Amplifications were carried out in 96-well plates in a CFX Connect Real-Time PCR Detection System (Biorad) with thermocycling conditions of 3 min at 95 °C, 20 s at 95 °C, and 30 s at 60 °C for 40 cycles. The quantification was reported as quantity relative to the internal reference.

### RNAse R digestion

RNAse R (Lucigen) was used to test the circular nature of the ambivirus genomes on CpAV1. Samples containing 2 µg of total RNA from infected *C. parasitica* ACP43 were incubated in RNAse R 1X reaction buffer, 1 U of RNAse R for 5, 15 or 30 minutes at 37 °C. Negative controls were obtained mixing 2 µg of total RNA with the reaction buffer without RNaseR and tested at time 0 and after 30 min of incubation at 37 °C. Reaction inactivation was performed adding the northern blot denaturation mix (see northern description above) to each samples and incubating at 65 °C for 20 min. Denatured samples were then analyzed through denaturing agarose gel electrophoresis and northern blotting as reported above.

### Deriving ambivirus-infected and ambivirus-free isogenic lines

Isolate -ACP34- was originally collected from a diseased tree (*Castanea sativa*) with evident cankers in Azerbaijan^13^. The *C. parasitica* isolates were maintained on PDA media at 4 °C.

Isolate ACP34 was inoculated in 90 mm Petri plates with PDA for two months and sixteen days with light/dark cycles of 12 h. Conidia were harvested from the culture by pipetting 10 ml of sterile water in the Petri plate for two minutes and scrubbing the mycelia twice at 1-min intervals. The spore suspension aliquot was collected and filtered with cotton placed in a 5 mL tip. Isogenic monoconidial isolates were obtained by serially diluting the harvested spore suspension and plating 300 mL of the diluted conidia in 150 mm Petri plates with serial dilutions (1:10^−1^ to 1:10^−10^). Single spore isolates were transferred after 5 days, and single fungal colonies were tested for ambivirus presence/absence by qRT-PCR. cDNA was synthesized with the High Capacity cDNA Reverse Transcription Kit (Appliedbiosystems, by Thermo Fisher Scientific) following the manufacturer’s protocol.

To diagnose CpAV1 in the isolates, qRT-PCR was performed using a 1700 fast SDS Real-Time PCR detection system (Applied Biosystems). The PCR reaction was performed in 10 μL using the I-Taq supermix (Biorad) and Taqman probe. Each 10 μL reaction mix contained 5 μL supermix, 0.15 μL probe, 4 μL of sterile water and 0.2 μL of forward and reverse primers (Supplementary Data 1), and 1 μL of cDNA. Amplifications were carried out in 96-well plates in a CFX Connect Real-Time PCR Detection System (Biorad) with thermocycling conditions of 3 min at 95 °C, 20 s at 95 °C, and 30 s at 60 °C for 40 cycles.

### In vitro culture on potato dextrose agar

After deriving the isogenic CpAV1-free and CpAV1-infected isolates, mycelia plugs were taken from the edges of subcultures of three isolates for each condition (+1, +4, +8, −2, −7, −12). The isolates were inoculated in 150 mm Petri plates and incubated in potato dextrose agar (PDA) (Sigma-Aldrich) at 26 °C for 14 days. For each isolate we included three biological replicates and mycelia diameters were measured for all isolates at three days intervals post-inoculation until the full diameter of the Petri plate is covered.

### Virulence characterization of virus-infected and virus-free isogenic isolates on live chestnut stem and apple fruits

Live chestnut stems of 30 cm were prepared for inoculating the isogenic CpAV1-free and infected isolates (1+, 4+, 8+, 2−, 7−, 12−). Cuttings to be used in the experiment were obtained from chestnut suckers from a chestnut orchard (European chestnut, *Castanea sativa* in Salò-BS, Italy). Each stem cutting was disinfected with 70% ethanol then the cuttings were coinfected with virus-positive and negative mycelia plugs at a 17 cm distance from each other. The experiment was performed with three biological replicates randomly distributed to have CpAV1-infected and CpAV1-free mycelia plug at the upper and lower side of the stems. A cork borer of 5 mm diameter was used to artificially displace a section of the bark. Mycelia plugs were then placed under the removed barks and tightly taped for wound closure. Also, negative controls without mycelia plugs but with similar wounds were made. The cuttings were stored in 1.5 L of vermiculite soaked with water for 21 days.

Apples (cv Delicious) were also used to verify virulence. Each apple was inoculated in 4 points with a CpAV1 infected and a CpAV1 free strain (2 replicates each on a single apple, in opposite positions). Inoculated apples were kept at room temperature for 14 days.

### Statistical analysis

Analyses of the data referred to the measures of lesion or canker size in the virulence assays (apples and chestnut cuttings) were performed with one-way analysis of variance (ANOVA) using the R-statistical program^39^. Significant differences were assessed with Tukey’s post hoc test (*p* < 0.05).

### Validation of RNA-only nature of viroid-like and delta-like elements in selected fungal metatranscriptomes

We screened HTS read libraries present in two bioprojects: PRJNA524447 corresponding to a collection of *Rhizoctonia solani* isolates^40^ and PRJNA629308 corresponding to an orchids and ericoid mycorrhizal fungi collection^12^ using the same pipeline described above. All the selected contigs were checked in each of the biological samples to track contig-positive samples using the primers in Supplementary Data 1 in a standard PCR reaction using OneTaq® DNA Polymerase (New England Biolabs, Ipswich, MA, USA). Control genes were used to check the possibility to amplify DNA from the total nucleic acid samples without the presence of inhibitory compounds. Obtained short PCR fragments were separated on 2% agarose gel in 1× TAE buffer.

### Reporting summary

Further information on research design is available in the Nature Portfolio Reporting Summary linked to this article.

## Supplementary information

Supplementary Information

Peer Review File

Description of Additional Supplementary Files

Supplementary Data 1

Reporting Summary

## Data Availability

All Serratus data are released into the public domain immediately in accordance with the International Nucleotide Sequence Database Collaboration (16) and freely available at https://github.com/ababaian/serratus/wiki/ambivirus_extended_data. Assembled genomes for this study are additionally available on GenBank under BioProject PRJNA951513. Source data are provided with this paper.

## Source data

Source Data File

## Materials availability

Biological materials, where available, are available upon request provided a MTA is signed. Tulasnella isolate MUT4048 and MUT4047 are Deposited in Mycotheca Universitatis Taurinensis (MUT), ACP 34 is a *Cryphonectria parasitica* isolate that is being deposited in a collection (accession numbers: MUT 6705 is ACP34 sc1; MUT 6706 ACP34 sc4; MUT 6707 ACP34 sc8; MUT 6708 ACP34 sc9; MUT 6709 ACP34 sc7) and is currently available upon request to co-author D.N.A. The ambivirus positive isolate used for the Northern blots and RACE experiments, ACP43, is deposited in Mycoteca Universitatis Taurinensis (MUT) as MUT6704.

## Code availability

Software created for this study are available at https://github.com/ababaian/serratus/tree/infernal-dev, https://github.com/rcedgar/circuclusthttps://github.com/rcedgar/palmscan, https://github.com/rcedgar/jupiter, and https://github.com/rcedgar/ribozy under the open-source GPLv3 license.
