# Supplementary material for: Hybrids of RNA viruses and viroid-like elements replicate in fungi
Source: Nat Commun. 2023 May 5;14:2591. doi: 10.1038/s41467-023-38301-2 (PMC10162972; doi:10.1038/s41467-023-38301-2)
Supplement: Supplementary file 1 — Supplementary Information [file 41467_2023_38301_MOESM1_ESM.pdf]

Supplementary Information for

Authors:

Marco Forgia[1†], Beatriz Navarro[2†], Stefania Daghino[1], Amelia Cervera[3], Andreas Gisel[4], Silvia Perotto[5], Dilzara N. Aghayeva[6], Mary Funmilayo Akinyuwa[7‡], Emanuela Gobbi[8], Ivan N. Zheludev[9], Robert C. Edgar[10], Rayan Chikhi[11], Massimo Turina[12*], Artem Babaian[13*], Francesco Di Serio[2*], Marcos de la Peña[3*]

Correspondence to: massimo.turina@ipsp.cnr.it; ab2788@cam.ac.uk; francesco.diserio@ipsp.cnr.it; rivero@ibmcp.upv.es

This PDF file includes:

Supplementary Note 1

Supplementary Fig. 1 to 9

Supplementary Table 1 to 2

Supplementary Note 1

<u>The linear form of the positive sense and negative sense genomes of ambiviruses are not complementary along the full-length segments</u>

All the Baltimore classes of RNA viruses (III, IV and V) contain viruses that replicate via RNA dependent RNA polymerases: in these classes these enzymes copy the full-length template linear genomic RNA in a full length antigenomic linear RNA for maintaining genomic RNA identity during replication. Presence of complementary full length genomic and antigenomic RNAs is, to our knowledge, without exception in RNA viruses and is a hallmark of their replication strategy. This implies that the 5' end of the antigenomic RNA is complementary to the 3' end of the genomic RNA, and often, when antigenomic RNA accumulation is abundant, the nt sequence of the 3' end of the genomic RNA can be inferred indirectly by sequencing the 5' end of the antigenomic RNA with Hirzmann's approach [38] .

Given that ambiviruses accumulate in fungi as virion-less entities, genomic and antigenomic RNAs are purely conventional and we follow a previously proposed convention where the positive sense (genomic) RNA is the one encoding for ORF-A, the putative RdRp, while antigenomic (negative) sense RNA is the one encoding for ORF-B [12]. Given these premises, we performed two distinct 5' RACE experiments using oligonucleotides close to the presumed 5' end of the genomic and antigenomic linear monomeric RNAs on three distinct ambiviruses (TuAmV1, TuAmV4 and CpAV1) in total RNA from infected fungal extracts (*Tulasnella spp.* and *Cryphonectria parasitica*) and in all three cases we had surprising results: the first 245 nt for CpAV1, 256nt for TuAmV4 and 223nt for TuAmV1 of the 5' of the genomic RNAs were exactly complementary to the first 245 nt for CpAV1, 256nt for TuAmV4 and 223nt for TuAmV1 5' of the antigenomic RNA. If genomic and antigenomic would be complementary along the full length, this would imply that the monomeric genomic and antigenomic have a same orientation sequence repeat of circa 250 nt (Supplementary Fig. 3, panel A), which would be unprecedented for RNA viruses. This prompted us to further investigate if such repeat indeed exists on the genomic and antigenomic RNA-

Attempts to sequence the 3' ends of ambivirus RNAs of both polarity strands failed, which is consistent with the cleavage by HHRz and HPRz motifs which leave a blocking 2'-3' cylic phosphate on the 3' termini [42].

Given that we failed to use alternative RACE methodology to determine more directly the 3' end of the genomic RNA, we proceeded to carry out RT-PCR amplification, that would use oligonucleotides to prime cDNA synthesis specific for the genomic or antigenomic RNAs. We selected for RT-qPCR amplification regions of the genome across the putative link between the conserved repeat and the specific sequences upstream (for determining the presence of the 3' putative repeat in the genomic RNA). As control, we included in the same assay the

quantification of the central region of the RNA genomic segment. Surprisingly, the ratio between the two is not consistent with the expected equimolar existence of the two repeats at the 5’ and 3’ end of the genomic RNA (Supplementary Fig. 3, panel B).

The same experiment carried out for the antigenomic RNA provided the same results: the repeat at the 3’ end is not present in the expected same concentration as the 5’ end (Supplementary Fig. 3, panel C). Minimal amount of the repeats is indeed present because in the same extracts we have shown accumulation of putative dimer or circRNAs of both strands, that would indeed provide some amplification.

In conclusion, the two monomeric forms of the genomic and antigenomic RNA are not full-length complementary RNAs, pointing to a replication mechanism for these RdRp-encoding RNAs different from that of the other Baltimore classes of RNA viruses.

These data are consistent with the different positions of the ribozyme self-cleaving sites in the genomic and antigenomic RNAs.

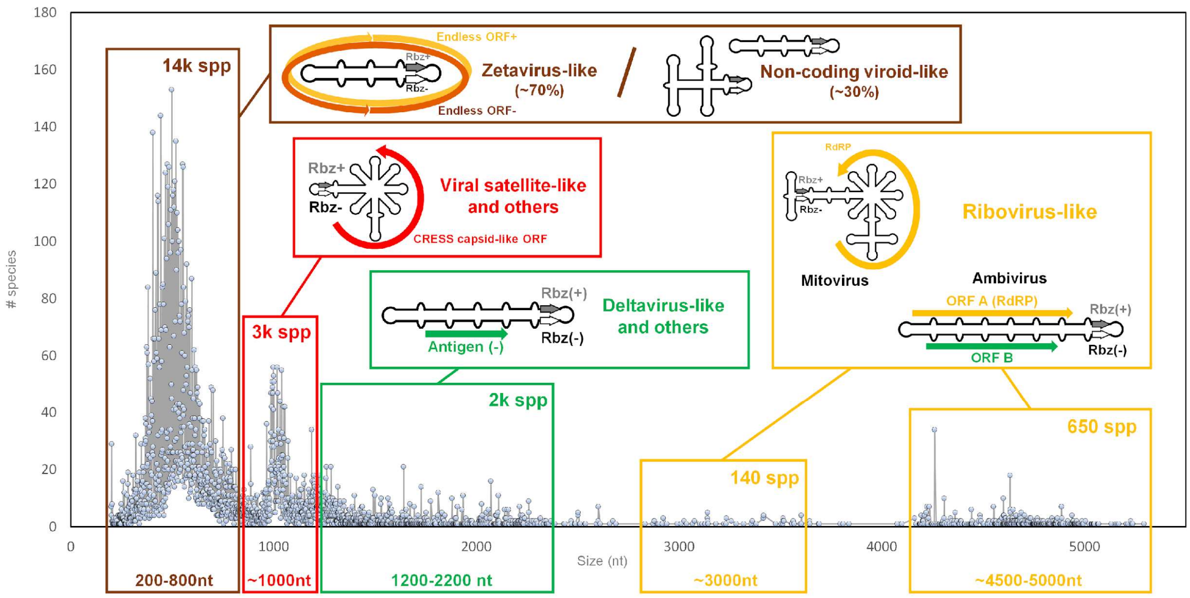

Supplementary Fig. 1. Size distribution of the circRNA genomes with two ambisense ribozymes. Distribution graph of the 20,364 detected species-like operational taxonomic units (sOTU) at 90% nucleotide sequence identity based on their nucleotide length. Representative examples of their genome organization and protein coding capabilities of the circRNAs from each of the 5 major groups are depicted in the insets. Most (~70%) of the small circRNAs (200-800 nt) detected in this study resemble the previously reported Zetavirus-like genomes, which are characterized by a rod-like conformation and potential capability of encoding endless ORFs (no stop codons) in either one or both polarities[14]. A smaller fraction (~30%) of the viroid-like genomes corresponded to putatively non-coding circRNAs. Among the medium sized genomes (800-2000 nt) we detected many examples of circRNAs encoding ORFs with sequence and/or structural homology to either the classical jelly-roll capsid of diverse RNA but also DNA viruses (Eukaryotic Circular Rep-Encoding Single-Stranded or CRESS DNA viruses) or the delta antigen of deltavirus-like (genomes depicted with a highly branched or a rod-like circRNA structure, respectively). Larger genomes (3,000-5,000 nt) mostly encode an RNA-dependent RNA Polymerase (RdRp) with sequence and/or structural similarity to fungal mitoviruses and ambiviruses RdRps (genomes depicted with a branched or a rod-like circRNA structure, respectively).

A

NGS bioproject	Host Isolate	Contig name	length
PRJNA524447	*R.solani* IBRS16, IBRS19	Z1	423
PRJNA524447	*R.solani* IBRS16, IBRS19	Z2	279
PRJNA524447	*R.solani* IBRS16, IBRS19	Delta	1152
PRJNA629308	Tulasnella sp. O9	11108	1261
PRJNA629308	Tulasnella sp. O10	38423	490
PRJNA629308	Tulasnella sp. O10	6686	1624
PRJNA629308	Tulasnella sp. O9	19938	1261

B

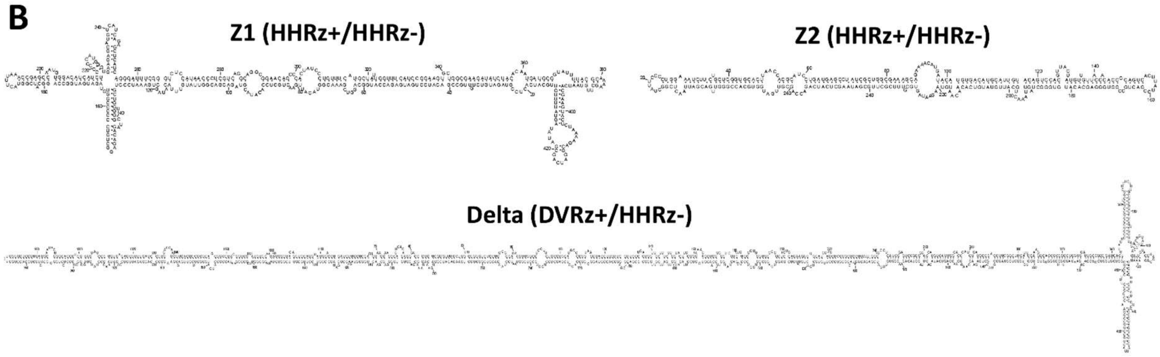

C

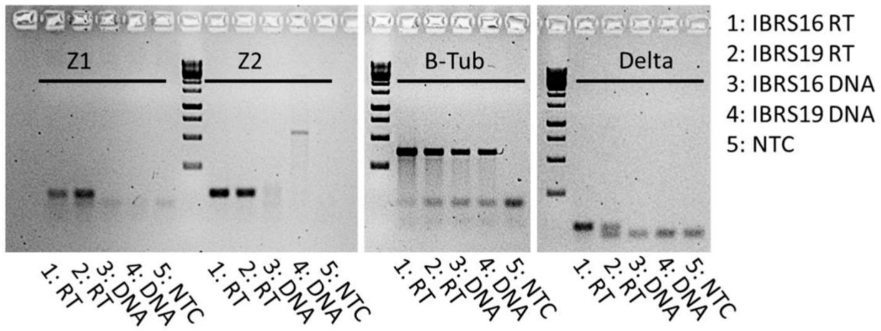

D

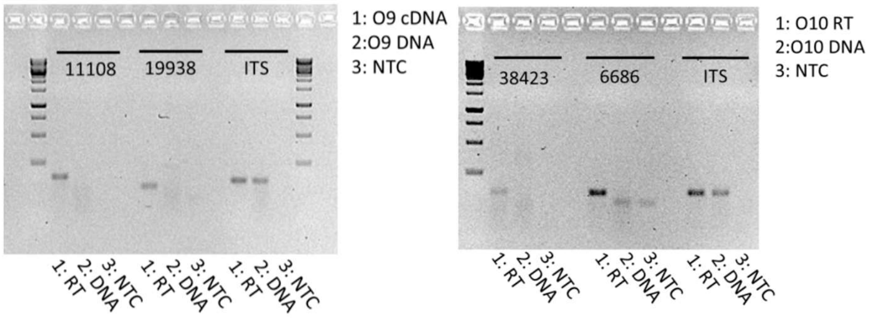

Supplementary Fig. 2. Members of the ribozycirculoma detected in fungal collections. (A) Table with circular contigs encoding two ambisense ribozymes detected in transcriptomic datasets from fungal collections of *Rhizoctonia solani* and *Tulasnella* spp. (B) Predicted secondary structure and (C) RT/PCR amplifications of the contigs detected in the *Rhizoctonia solani* library. The results were obtained from two independent experiments. (D) RT/PCR amplification of the circular contigs from different *Tulasnella spp.* isolates from the orchid mycorrhizal fungi library. For each fungal isolate, a nuclear gene is tested as a control on the DNA sample (B-Tubulin gene on *R. solani* and *Tulasnella* ITS for orchid fungi). Molecular weight marker corresponds to the GeneRuler 1 kb DNA Ladder (Thermo Fisher Scientific, Ref. SM0312)

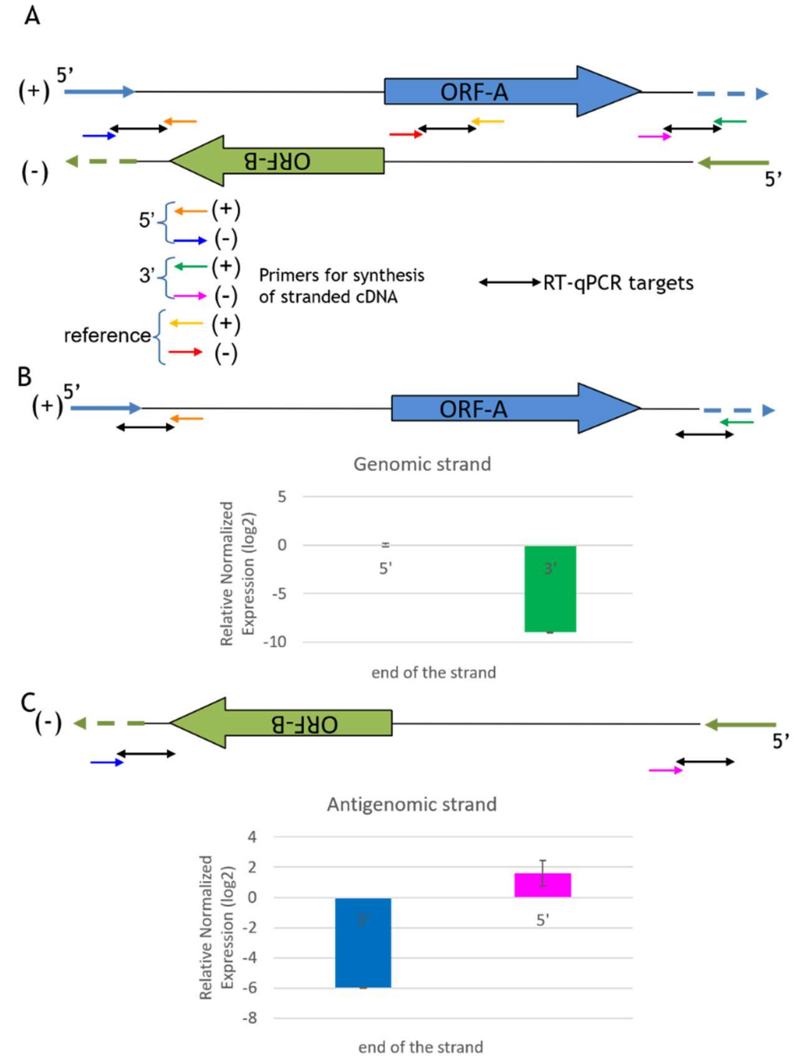

Supplementary Fig. 3. The linear replication intermediates of both polarity strands are asymmetrical. (**A**): RACE analysis of 5' ends of genomic (+) and antigenomic (-) RNA of TuAmV1 revealed 222 nt complementary sequences (thin solid arrows): broken arrows correspond to putative same sense repeats in the genomic and antigenomic segment. Thinnest colored and black arrows respectively correspond to strand specific oligonucleotides and to amplification products across the putative junctions between the sequence repeats and the specific region of the genomic and antigenomic RNA, or on the ORF-A coding sequence (red and yellow arrows) as an internal reference for the abundance of the genomic and antigenomic strands. (**B**): relative quantification (log2) of the genomic strand RNA region across the junction of the 5' and 3' repeat. (**C**): relative quantification (log2) of the antigenomic strand RNA region across the junction of the 5' and 3' repeat. The color of the bars reflects the oligonucleotide used for the cDNA synthesis and represented by the thinnest arrows. In the 5' end region of the genomic, the accumulation orders of magnitude larger than the putative 3' end of the genomic RNA. For the antigenomic RNA the 5' end accumulates more than the 3' end. For both the genomic and the antigenomic strands the accumulation of the 5' region is comparable to the one of the internal reference region on the ORF-A.

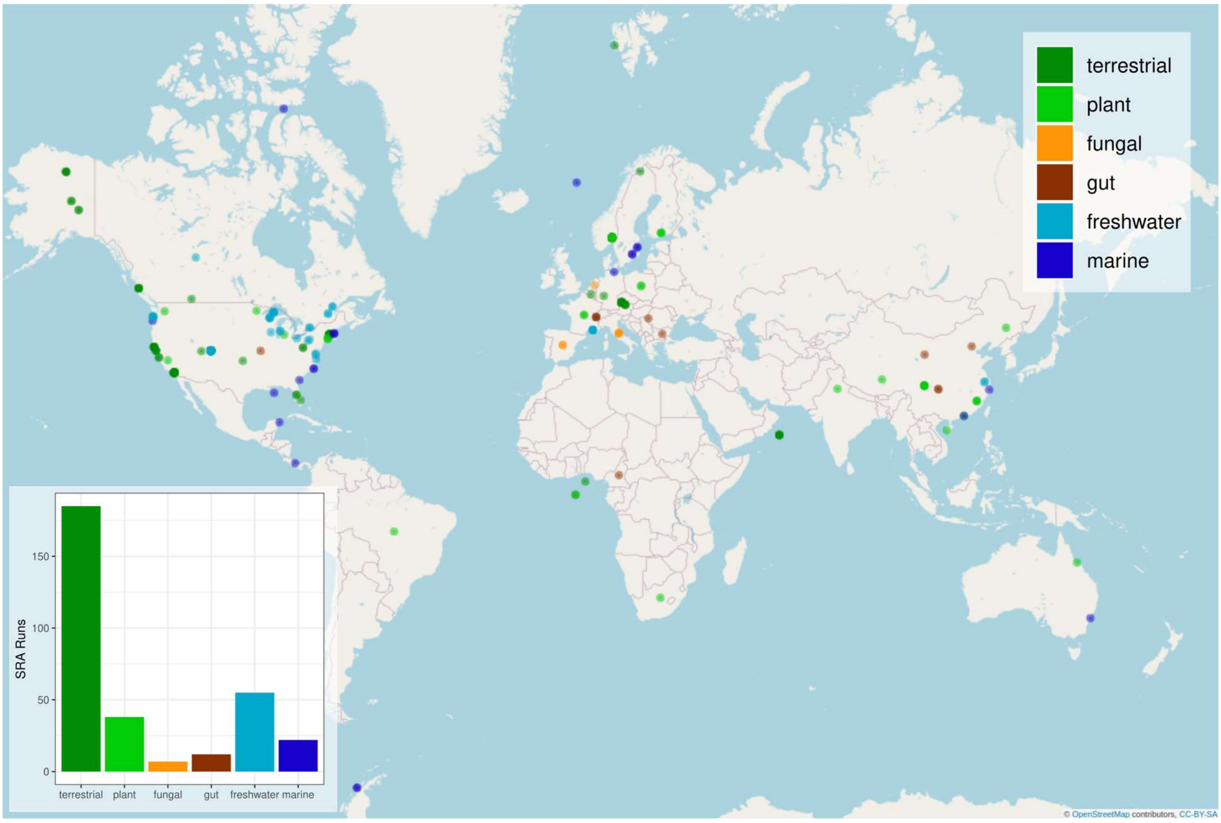

Supplementary Fig. 4. Ambiviruses genomes were detected in datasets from geographical and ecological diverse sources. Ambiviruses genomes were identified in 320 sequencing datasets of the Sequence Read Archive (SRA). Each dataset was categorized based on its source environment (terrestrial, gut, freshwater or marine) or source material (plant or fungal), and geospatial coordinates were extracted from sample metadata or inferred from the associated metadata and/or study descriptions. Map image is open data obtained from OpenStreetMap

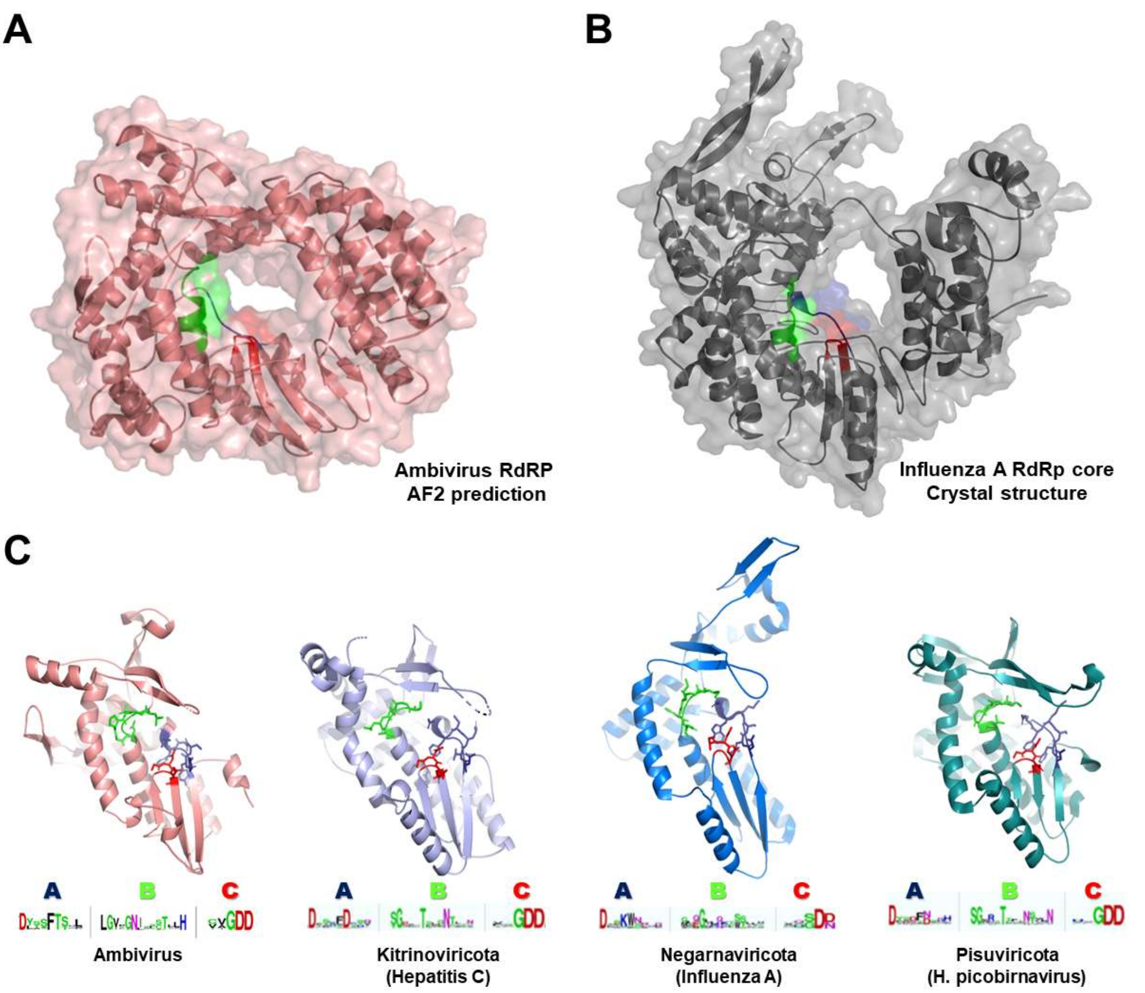

Supplementary Fig. 5. Structural models for the RNA-dependent RNA polymerases. Structural models for the RNA-dependent RNA polymerases (RdRp) of (**A**) the Armillaria borealis ambi-like virus 2 (Accession MW423810.1, as predicted by ColabFold-AlphaFold2 algorithm) and (**B**) the bat influenza A RdRp core (PDB 6t0u, chain B, as obtained by cryoelectron microscopy [43] show a high global similarity (Z score 19.2, rmsd 4.0 A) and equivalent placement of the putative catalytic residues of the palm motif (A box in blue, B box in green and C box in red). (**C**) Structural comparison of the RdRp palm domains of the *Armillaria borealis* ambivirus 2 (Alphafold2 predicted, see A) and those experimentally determined for Hepatitis C (PDB 3br9), Influenza A (PDB 4wsb) and human picobirnavirus (PDB 5i61) RdRps. Highly conserved catalytic residues for each palm motif are shown as sticks. Consensus sequences of the motifs A, B and C for each viral phylum are shown at the bottom.

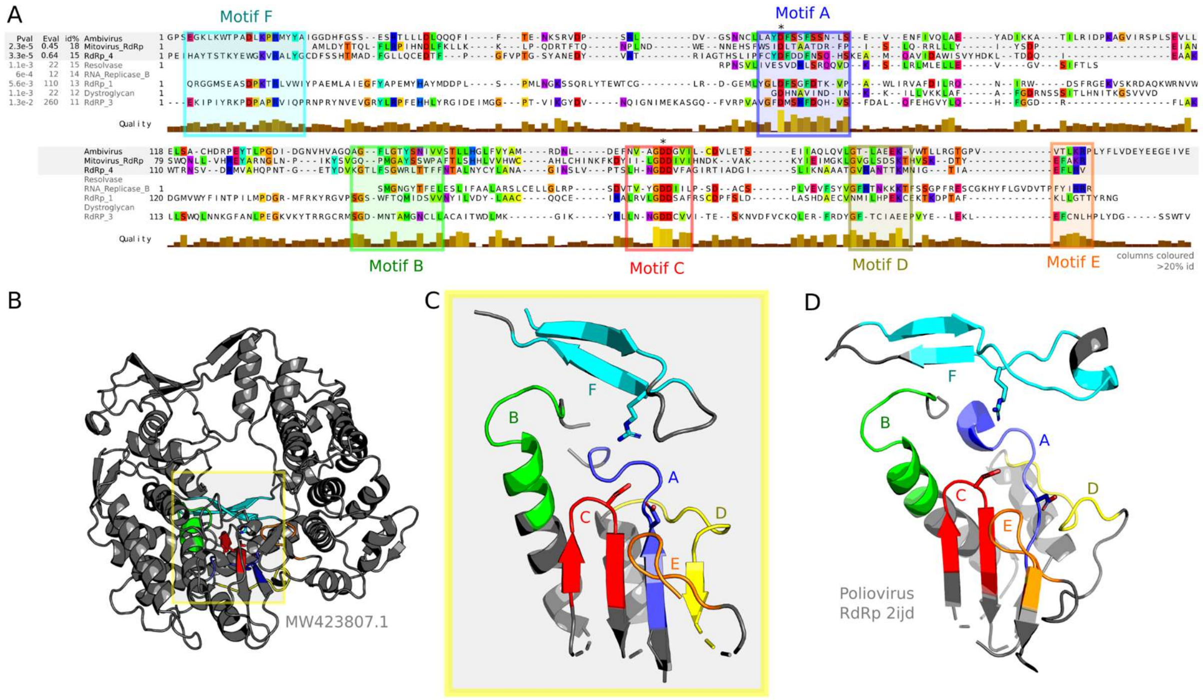

Supplementary Fig. 6. Deep homology of Ambivirus RNA-dependent RNA Polymerase. (**A**) Multiple sequence alignment (MSA) of all available (n = 442) ambivirus ORF-A (the hypothesized RNA dependent RNA polymerase, RdRp) was used to construct a hidden Markov model (HMM) profile. Deep homology was tested by a HMM profile-profile alignment (hhpred[44]) against PFAM-A (v35) HMM models. Significant matches (p-value <0.05; E-value <1) to Mitovir_RNA_pol (PF05919.14) and RdRp_4 (PF02123.19) were identified, which correspond to the mitovirus-specific and lueteovirus/totivirus-specific RdRp models, respectively. MSA of representative sequences from each HMM are shown with conserved motif A, B, C, D, E and F highlighted. (**B**) ColabFold structure prediction of the RdRp from Amillaria borealis Ambivirus 2 (MW423807.1) shows characteristic right-hand palm organization and (**C**) the analogous orientation of the conserved motifs. (**D**) RdRp conserved motifs from poliovirus crystal structure (pbd: 2ijd) shown for comparison.

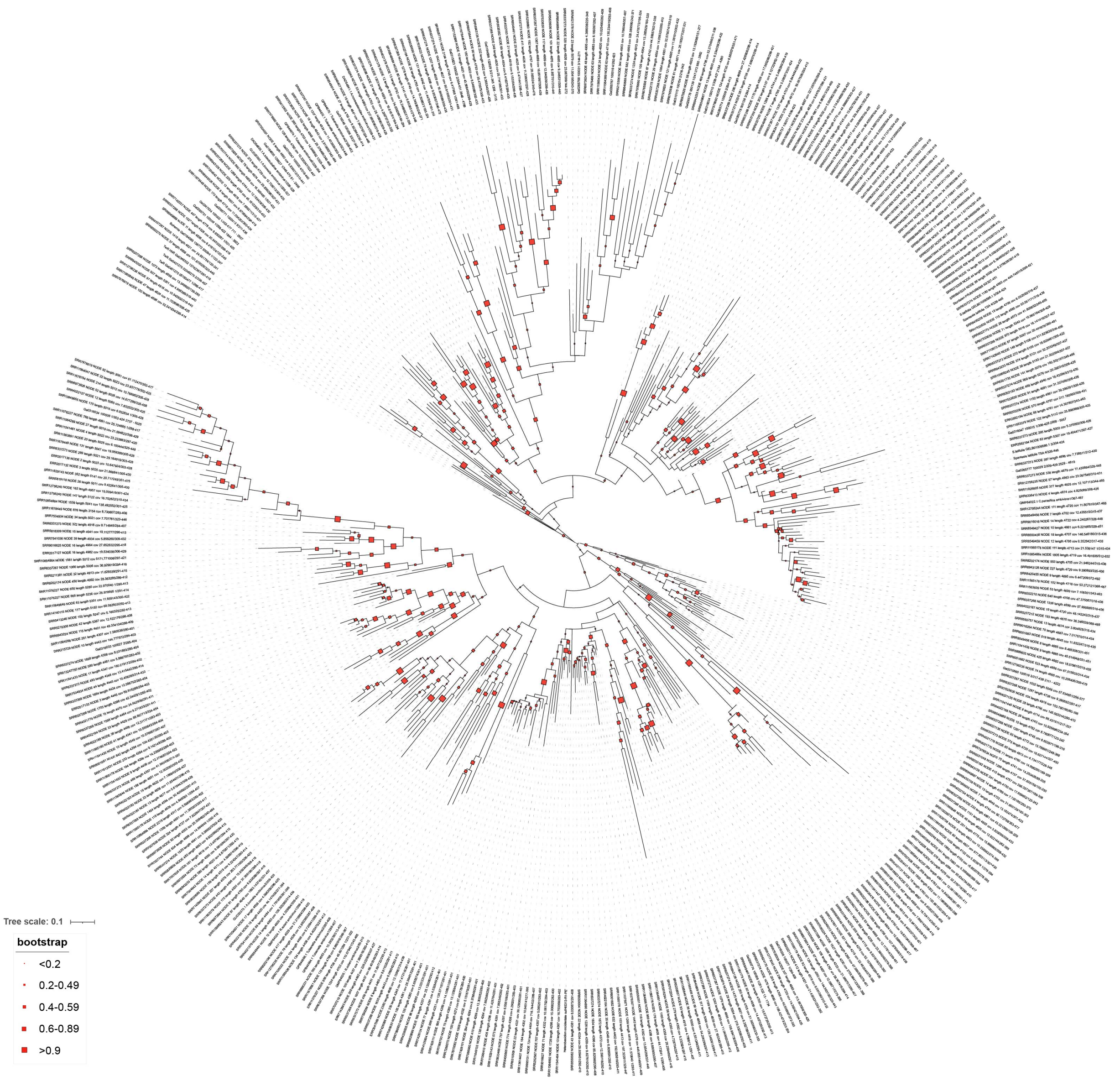

Supplementary Fig. 7. Phylogram for Ambiviruses. Maximum-likelihood phylogenetic tree of the RNA-dependent RNA polymerase palm domains from the 439 distinct species-like operational taxonomic units (sOTUs) of known ambiviruses with bootstrap values. The phylogeny was built with the Jones-Taylor-Thornton (jtt) substitution model based on 168 alignment sites.

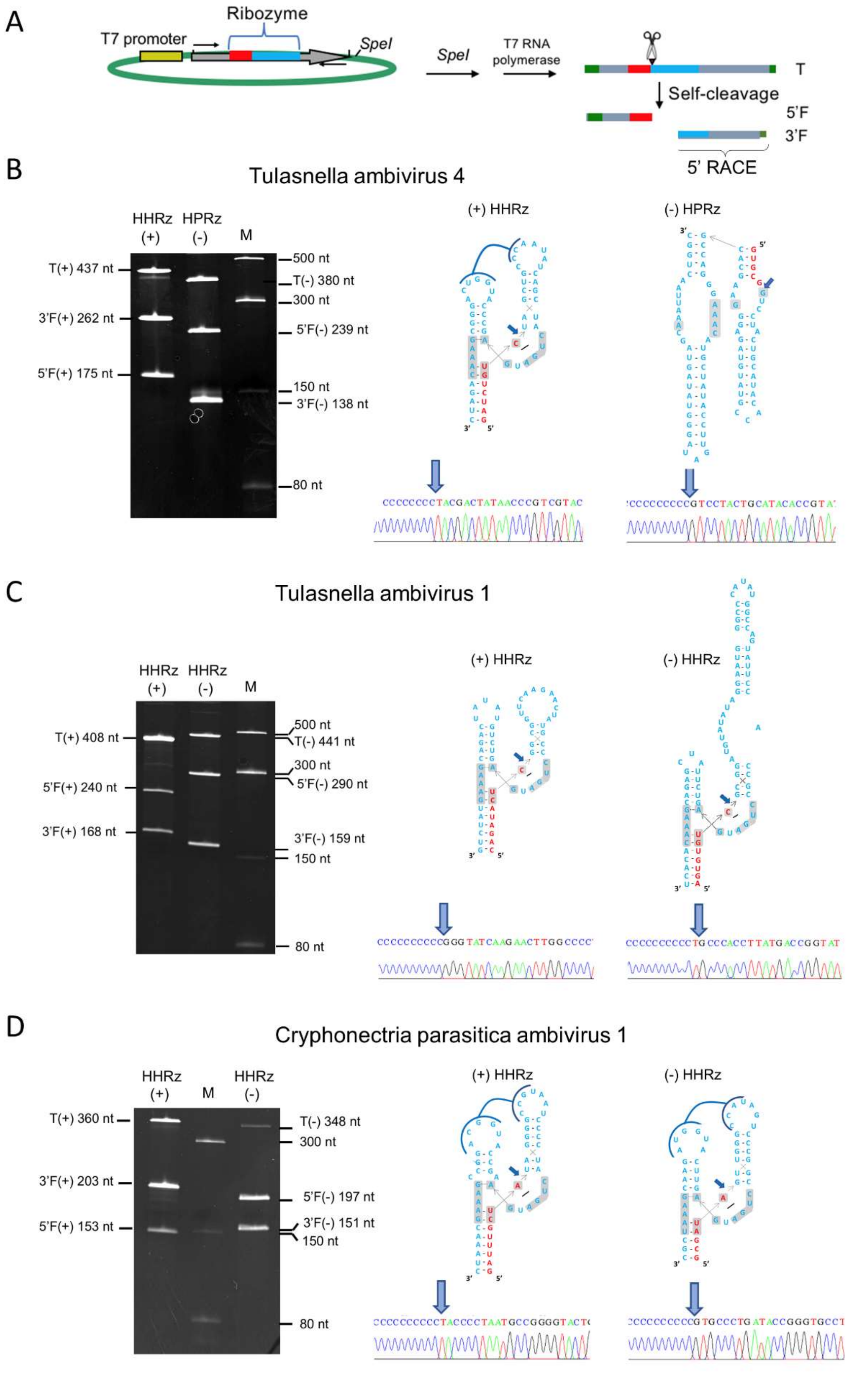
A
Ribozyme
T7 promoter
SpeI
SpeI
T7 RNA polymerase
T
Self-cleavage
5'F
3'F
5' RACE
B
Tulasnella ambivirus 4
HHRz (+)
HPRz (-)
M
T(+) 437 nt
3'F(+) 262 nt
5'F(+) 175 nt
500 nt
T(-) 380 nt
300 nt
5'F(-) 239 nt
150 nt
3'F(-) 138 nt
80 nt
(+) HHRz
(-) HPRz
C
Tulasnella ambivirus 1
HHRz (+)
HHRz (-)
M
T(+) 408 nt
5'F(+) 240 nt
3'F(+) 168 nt
500 nt
T(-) 441 nt
300 nt
5'F(-) 290 nt
3'F(-) 159 nt
150 nt
80 nt
(+) HHRz
(-) HHRz
D
Cryphonectria parasitica ambivirus 1
HHRz (+)
M
HHRz (-)
T(+) 360 nt
3'F(+) 203 nt
5'F(+) 153 nt
T(-) 348 nt
300 nt
5'F(-) 197 nt
3'F(-) 151 nt
150 nt
80 nt
(+) HHRz
(-) HHRz

Supplementary Fig. 8. Ribozymes contained in ambivirus RNAs are active *in vitro.* (**A**) Schematic representation of the DNA template for the *in vitro* transcription, consisting of a plasmid containing the cDNA of the self-cleaving ribozyme to be tested, and of the expected RNA products. Plasmids containing the ribozyme sequences of Tulasnella ambivirus 4 (TuAmV4), Tulasnella ambivirus 1 (TuAmV1) and Cryphonectria parasitica ambivirus (CpAV1) were linearized and transcribed with T7 RNA polymerase to produce full-length transcripts (T) and the respective 5' and 3' fragments (5'F and 3'F, respectively) derived from the ribozyme self-cleaving activity. In green, plasmid sequences; in yellow, polymerase promoter; in grey ambivirus RNA sequences with 3'F and 5'F ribozymatic regions denoted in blue and red, respectively. The predicted self-cleavage site is indicated by an arrowhead. (**B**-**D**) Left panels, analyses by denaturing 5% PAGE of the *in vitro* transcription products of the plasmids containing the cDNAs of the self-cleaving ribozymes identified in the (+) and (-) polarity strands of the genomic RNAs of TuAmV4, TuAmV1 and CpAV1. The sizes of the full-length, 3'F and 5'F RNAs generated during each transcription were consistent the self-cleaving activity of the ribozyme in each transcript. M, RNA ladder with sizes indicated on the left (300, 150 and 80 nt); numbers on the right and on the left indicate the size of the RNAs. Middle and right panels, primary and secondary structure of (+) and (-) ribozymes contained in the viral genomic RNAs, respectively, of TuAmV4 (B), TuAmV1 (C) and CpAV1 (D). Self-cleavage site of each ribozyme was confirmed by 5' RACE of the 3'F fragment, with the respective sequencing electropherograms reported on the bottom. The same results were obtained from three independent experiments. The predicted self-cleavage site is indicated by a blue arrow. The nucleotides of the catalytic core conserved in most natural HHRz and HPRz structures are reported on a grey background.

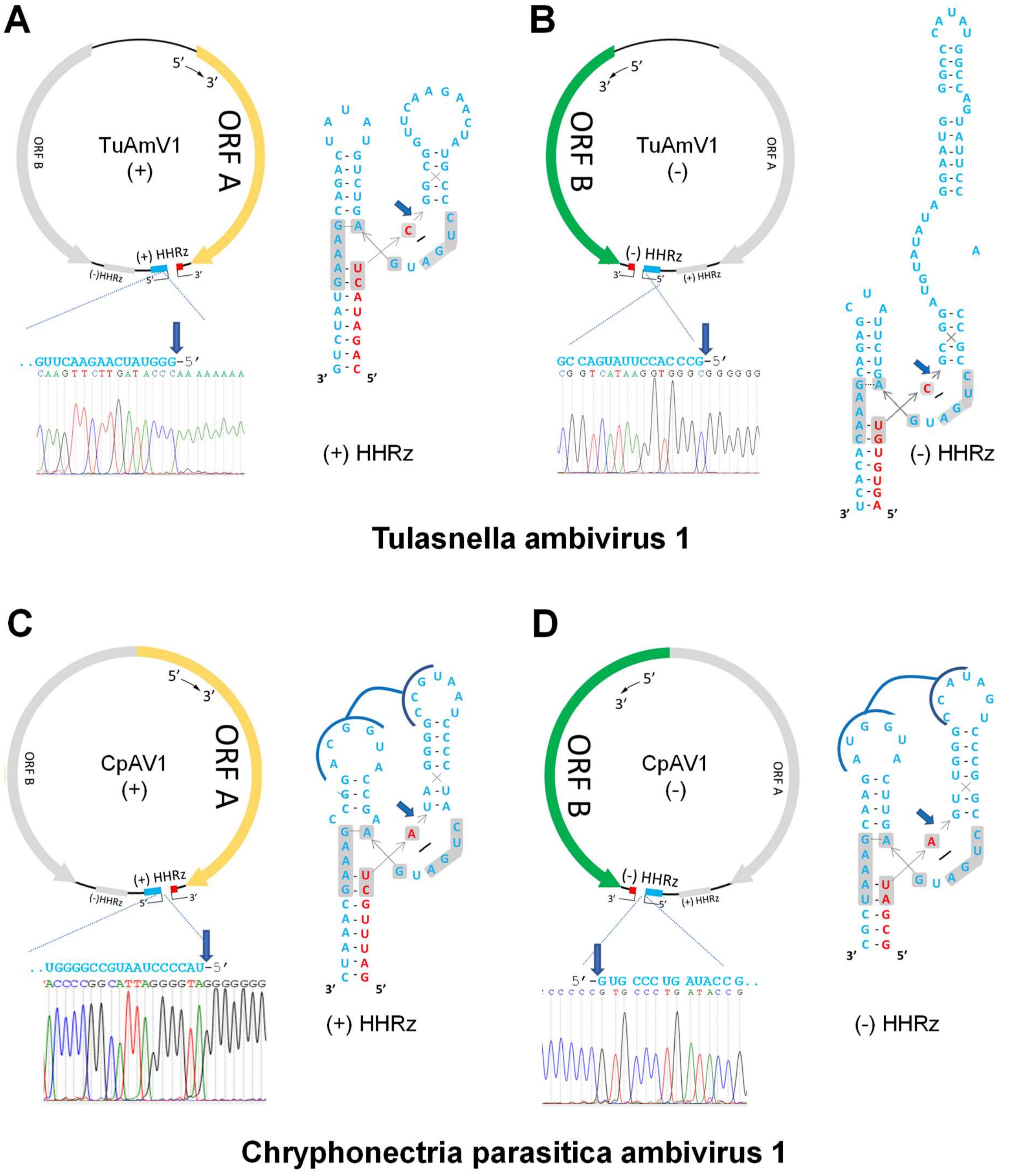

Supplementary Fig. 9. The 5′ terminal end of (+) and (-) genomic RNAs of both polarity strands of Tulasnella ambivirus 1 (TuAmV1) and Cryphonectria parasitica ambivirus 1 (CpAV1) are coincident with those predicted to be generated by the RNA self-cleavage mediated by the respective encoded ribozymes. (**A** and **B**) refer to (+) and (-) polarity strands of the genomic RNA of TuAmV1, respectively. (**C** and **D**) refer to (+) and (-) polarity strands of the genomic RNA of CpAV1, respectively. In each Panel: Left, up, schematic representation of the (+) (A and C) and the (-) (B and D) genomic RNAs of TuAmV1 and CpAV1, respectively, with the encoded ORF A and HHRz depicted in yellow and red/light-blue for the (+), and the encoded ORF B and HHRz depicted in green and red/light-blue for the (-) polarity strand; the position of the ORF and the ribozyme contained in the opposite polarity are reported in

grey. Left bottom, electropherogram of the sequenced 5' RACE products showing the 5' terminal end of each viral RNA, which is coincident with the one predicted based on the self-cleavage mediated by the respective encoded HHRz. Right: secondary structure of the HHRz contained in each viral RNA polarity strand; the predicted self-cleavage site is indicated by a blue arrow with the sequences at the resulting 3' and 5' terminal ends depicted in red and light blue, respectively. Nucleotides conserved in most natural HHRzs are reported on a grey background.

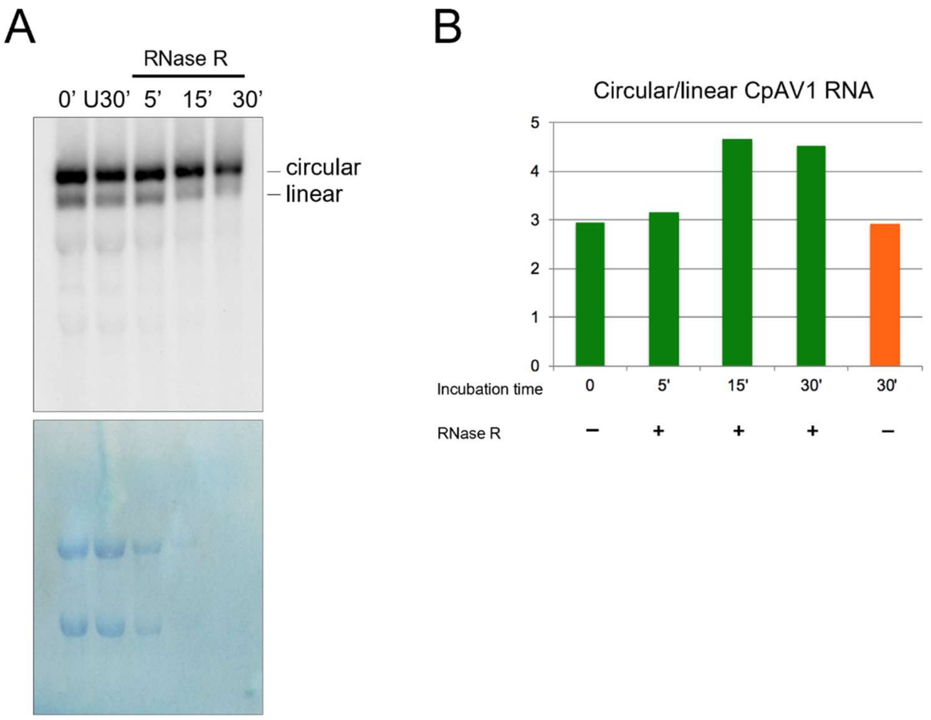

Supplementary Fig. 10. Circularity of ambivirus RNAs assessed by RNase R treatment. RNA preparations (2 ug) from *Cryphonectria parasitica* infected by CpAV1 were incubated with RNase R (1 UNIT) during 5, 15 and 30 min at 37°C in buffer supplied by the manufacturer. Controls at time 0 and at 30 min were incubated in the same conditions without RNase R. (**A**) Northern blot hybridization of treated RNA preparations with a specific Dig-RNA probe detecting the (-) polarity of CpAV1 (upper panel). Ribosomal RNAs were visualized on the membrane by toluidine blue staining (bottom Panel). While linear forms were almost completely degraded after incubation with RNase R for 30 min, the circular forms clearly resisted. No major changes of both the linear and circular RNA forms were observed after 30 min of incubation in the absence of RNase R. (**B**) Relative accumulation of circular/linear RNA forms of CpAV1 at different incubation times with RNase R based on the quantification of the Northern-blot hybridization signals. While in the absence of RNase R the ratio circular/linear RNAs does not change after 30 min incubation time (orange bar), such a ratio increases in the presence of RNase R at the different time points, showing a higher resistance of the circular forms. The same results were obtained from three independent experiments.

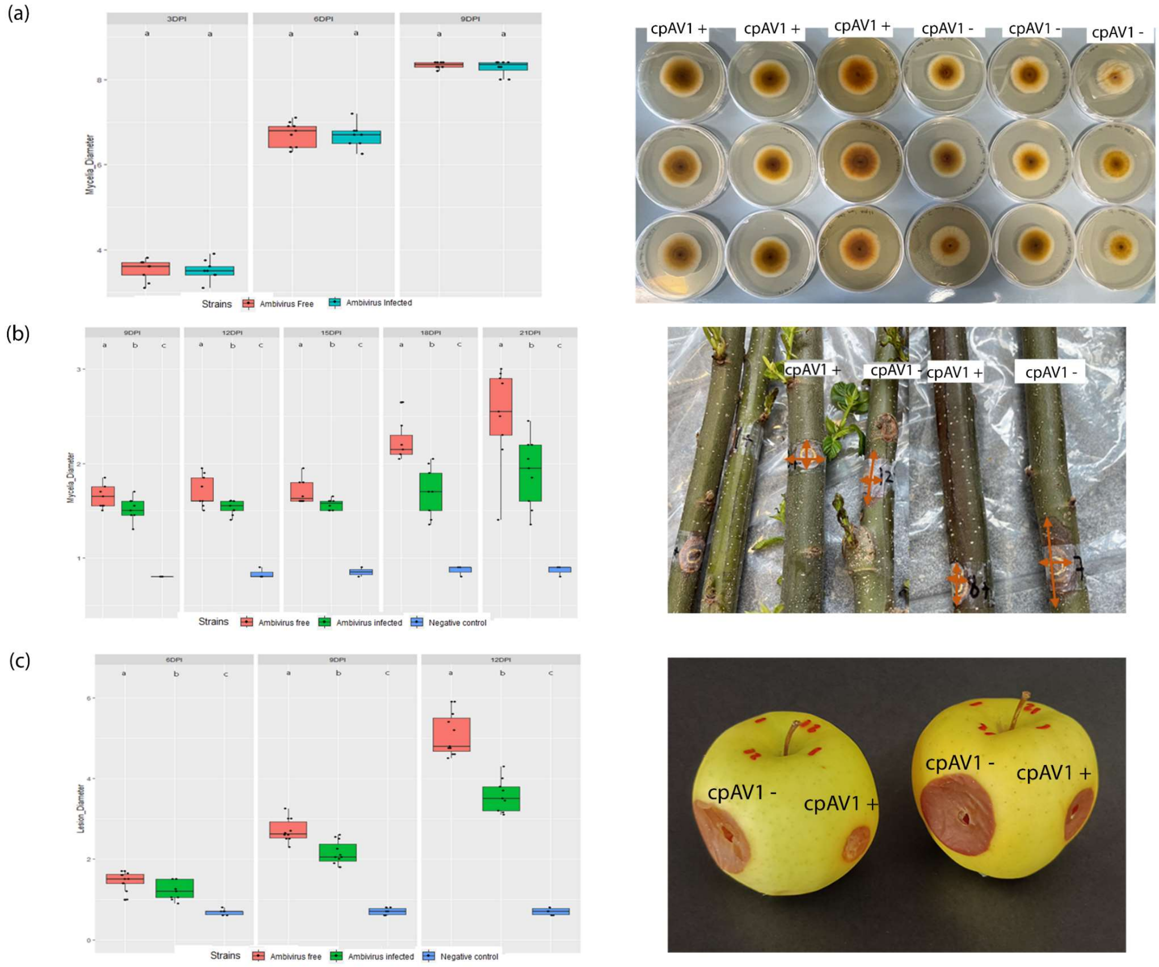

Supplementary Fig. 11. Cryphonectria parasitica ambivirus 1 (CpAV1) causes hypovirulence on chestnut cuttings and apples but has no effect on phenotype and growth rate in axenic potato dextrose agar cultures. (**A**) In vitro culture of ambivirus-infected and ambivirus-free *Cryphonectria parasitica* isolates in PDA (Sigma) at 7dpi; (**B**) Cankers caused by CpAV1-free and CpAV1-infected isolates at 9, 12, 15, 18 and 21 (picture) days post-inoculation. (**C**) Lesion diameters on apples at 6, 9 and 12 (picture) dpi; each apple displays two inoculated sites with a virus-positive isolate, and two inoculated sites with a virus-negative isolate. In (B) and (C), orange arrows represent diameters. The left side of each panel displays violin plots of measurements, the right-side exemplary photographs of the experiment. Each boxplot depicts the interquartile range (middle 50% of the data), the lower and upper edge shows the first and third quartile (25th and 75th percentile respectively), median (horizontal line within box), and sample numbers are portrayed by each black points. The whiskers are the contributions within the 1.5 interquartile range. The virulence experiment on apples and the growth experiment on PDA was repeated three times, whereas the chestnut cuttings virulence experiment was carried out once; all the experiments were carried out with 9 biological replicates for each treatment (virus-infected, virus-free). Source data are provided as a Source Data file.

Supplementary Table 1.

Ribozymes identified in ambivirus sequences available in GenBank.

Ambivirus	Ribozyme (+)*	Ribozyme (-)*	Accession number
Armillaria ambivirus 3	HHRz (286-340)	HHRz (4518-4523;1-75)	MW423812.1; **MW423813.1;** MW423811.1
Armillaria borealis ambi-like virus 1	HHRz (527-578)	HPRz (61-139)	MW423804.1 §
Armillaria borealis ambi-like virus 1	HHRz (526-577)	HPRz (60-138)	MW423805.1 §
Armillaria borealis ambi-like virus 2	HHRz (284-342)	HHRz (4524-4529;1-75)	**MW423806.1**; MW423807.1;MW423808.1; MW4238010.1§
Armillaria borealis ambi-like virus 2	HHRz (284-342)	HHRz (4520-4525;1-75)	MW423809.1 §
Armillaria ectypa ambi-like virus 1	HHRz (4952-4989**;1-19**)	HPRz (4466-4545)	BK014418.1 §
Armillaria luteobubalina ambi-like virus 1	incomplete genome	incomplete genome	BK014419.1 §
Armillaria mellea ambi-like virus 1	HPRz (2206-2310)	HPRz (2623-2548)	BK014420.1 §
Armillaria mellea ambi-like virus 2	HHRz (45-99)	HHRz (4226-4303)	BK014421.1§
Ceratobasidium ambivirus 1	HHRz (2390-2449)	HPRz (2568-2657)	MN793993.1
Cryphonectria parasitica ambivirus 1	HHRz (4613-4623; 1-49)	HHRz (196-249)	MT354566**
Heterobasidion ambi-like virus 1	HPRz (509-610)	HPRz (52-153)	MZ502384.1§
Heterobasidion ambi-like virus 2	incomplete genome	incomplete genome	MZ502385.1§
Heterobasidion ambi-like virus 3	HHRz (45-105)	HPRz (4725-4794)	MZ502386.1 §

Heterobasidion ambi-like virus 4	HHRz (273-324)	HPRz (4886-4913; 1-35)	MZ502387.1§
Phlebiopsis gigantea ambi-like virus 1	HPRz (582-685)	HPRz (76-183)	MZ448624.1 §
Rhizoctonia solani ambivirus 1	HHRz (2270-2327)	HHRz (2465-2548)	MT354567.1
Rhizoctonia solani ambivirus 2	HHRz (1470-1527)	HHRz (1601-1695)	MT354568.1
Tulasnella ambivirus 1	HHRz (4517-4578)	HHRz (1-19;4673-4736)	MN793991.1
Tulasnella ambivirus 2	likely incomplete genome	HHRz (121-209)	MN793992.1
Tulasnella ambivirus 3	HHRz (82-139)	HPRz (262-343)	MN793994 ***
Tulasnella ambivirus 4	HHRz (4903-4924;1-38)	HPRz (157-247)	MN793995.1
Tulasnella ambivirus 5	HHRz(4610-4632;1-34)	HPRz (191-269)	MN793996.1

*Numbers in brackets indicate the nt positions of the region spanning the ribozyme and refer to the virus sequence with the reported accession number. The (+) polarity is defined as the RNA strand coding for the polymerase (ORFA). Sequence available in GenBank as negative strand are marked by §. HPRzs are denoted in red.

** This sequence includes a terminal repeat of 243 nt, therefore the size of monomeric genomic RNA (excluding such a repeat) is 4623 nt.

** *This sequence includes a terminal repeat of 249 nt, therefore the the size of monomeric genomic RNA (excluding such a repeat) is 4869 nt.

Supplementary Table 2. Examples of Mito-like viruses showing putative circular genomes (based on k-mer repeats) and paired-ambisense ribozymes (based on InfeRNAl searches).

Entry	Name	Size (nt) circ	Ribozyme (+)	Ribozyme (-)
MW648461.1	Grapevine-associated mitovirus 14	3210	Twister	Hammerhead
MW648460.1	Grapevine-associated mitovirus 13	2968	Twister	Twister
MZ969057.1	*Fusarium asiaticum* mitovirus 7	2992	Varkud Satellite	Varkud Satellite
MZ969058.1	*Fusarium asiaticum* mitovirus 8	3038	Varkud Satellite	Varkud Satellite
SRR13754975	NODE_4086_length_2968_cov_23.415553	2919 circRNA	Twister	Twister
SRR13754975	NODE_3725_length_3056_cov_75.035584	3007 circRNA	Hammerhead	Twister
SRR13754975	NODE_4217_length_2942_cov_119.153474	2893 circRNA	Twister	Hammerhead
SRR6943136	NODE_404_length_3356_cov_47.364606	3307 circRNA	Twister	Twister
SRR6943136	NODE_433_length_3356_cov_47.808407	3307 circRNA	Twister	Twister
SRR6031102	NODE_2044_length_3642_cov_403.108714	3593 circRNA	Twister	Twister
SRR6765931	NODE_243_length_3085_cov_22.068061	3036 circRNA	Twister	Twister
SRR10849745	NODE_851_length_3256_cov_13.070688	3207 circRNA	Varkud Satellite	Varkud Satellite
SRR11565847	NODE_733_length_2946_cov_27.405499	2897 circRNA	Hammerhead	Hammerhead
SRR11565844	NODE_1557_length_2946_cov_62.985033	2897 circRNA	Hammerhead	Hammerhead
SRR12756234	NODE_868_length_3013_cov_19.082993	2964 circRNA	Twister	Twister
SRR11076227	NODE_2923_length_3393_cov_16.577711	3344 circRNA	Varkud Satellite	Varkud Satellite
SRR11541436	NODE_36_length_3163_cov_14.808927	3114 circRNA	Varkud Satellite	Varkud Satellite
SRR11565434	NODE_346_length_3332_cov_11.710341	3283 circRNA	Varkud Satellite	Varkud Satellite
SRR11565436	NODE_673_length_3332_cov_331.066278	3283 circRNA	Varkud Satellite	Varkud Satellite
SRR12610547	NODE_89_length_3256_cov_6.980522	3207 circRNA	Varkud Satellite	Varkud Satellite
SRR7142037	NODE_1346_length_3408_cov_590.197601	3359 circRNA	Varkud Satellite	Varkud Satellite

42 Takagi, Y., Ikeda, Y. & Taira, K. Ribozyme mechanisms. *New Aspects in Phosphorus Chemistry Iv* **232**, 213-251 (2004). https://doi.org:10.1007/b13783

43 Wandzik, J. M. *et al.* A Structure-Based Model for the Complete Transcription Cycle of Influenza Polymerase. *Cell* **181**, 877-893 (2020). https://doi.org:10.1016/j.cell.2020.03.061

44 Söding, J., Biegert, A. & Lupas, A. N. The HHpred interactive server for protein homology detection and structure prediction. *Nucleic Acids Research* **33**, W244-W248 (2005). https://doi.org/10.1093/nar/gki408
