# Supplementary material for: Hybrids of RNA viruses and viroid-like elements replicate in fungi
Source: Nat Commun. 2023 May 5;14:2591. doi: 10.1038/s41467-023-38301-2 (PMC10162972; doi:10.1038/s41467-023-38301-2)
Supplement: Supplementary file 3 — Description of Additional Supplementary Files [file 41467_2023_38301_MOESM3_ESM.pdf]

Description of Additional Supplementary Files:

Supplementary Data 1: Oligonucleotides used in this study and their features
